# Structural basis of isoform-specific cAMP regulation and autoinhibition in hyperpolarization-activated cyclic nucleotide-gated channels

**DOI:** 10.1016/j.jbc.2026.113589

**Published:** 2026-09-23

**Authors:** Valentina Zorzini, Jessica Kleiz-Ferreira, Marijke Brams, Rodrigo Gallardo, Steven De Gieter, Jana Kusch, Chris Ulens

**Affiliations:** 1 Laboratory of Structural Neurobiology, Department of Cellular and Molecular Medicine, Faculty of Medicine, KU Leuven, Leuven, Belgium; 2 School of Molecular and Cellular Biology, Faculty of Biological Sciences and Astbury Centre for Structural Molecular Biology, University of Leeds, Leeds, United Kingdom; 3 Center for Structural Biology, Vlaams Instituut voor Biotechnologie, Brussels, Belgium; 4 Structural Biology Brussels, Department of Bioengineering Sciences, Vrije Universiteit Brussel, Brussels, Belgium; 5 Institut für Physiologie II, Universitätsklinikum Jena, Jena, Germany

**Keywords:** ion channel, hyperpolarization-activated channel, cyclic AMP, cAMP, HCN channel, structural biology, cryo-EM, electrophysiology

## Abstract

Hyperpolarization-activated, cyclic nucleotide-gated (HCN) channels generate rhythmic electrical activity in cardiac and neuronal tissues, with isoform-specific cyclic AMP (cAMP) sensitivity remaining poorly understood. While HCN2 exhibits strong cAMP regulation, HCN1 shows minimal response. To investigate the structural basis of this divergence, we analyzed two engineered HCN1 variants using cryo-electron microscopy. One variant (HCN112) incorporates the C-linker and cyclic nucleotide-binding domain from HCN2 into the HCN1 backbone and exhibited enhanced cAMP sensitivity, with structural analysis revealing a compressed cytoplasmic domain arrangement that may facilitate regulatory interactions. In contrast, the truncated HCN1ΔC variant (lacking the cytoplasmic domain) displayed an intermediately open pore conformation, supporting auto-inhibitory regulation by the cyclic nucleotide-binding domain in wild-type channels. These structural insights elucidate how domain-specific interactions modulate cAMP-dependent gating and intrinsic auto-inhibition, providing insights into questions about mechanistic divergence among HCN isoforms. Our findings not only shed new light on the structural mechanisms underlying isoform-specific cAMP sensitivity but also have implications for the development of therapeutic strategies targeting HCN channels in neurological and cardiac disorders.

Ion channels are integral membrane proteins that act as molecular switches of electrochemical signaling in the central nervous system (1). More specifically, the Hyperpolarization-activated Cyclic Nucleotide-sensitive (HCN) channels belong to a family of cation channels that is activated by hyperpolarizing potentials and regulated by cyclic AMP (cAMP). HCN channels serve as nonselective voltage-gated cation channels that contribute to spontaneous rhythmic activity in the plasma membranes of heart and brain cells. This type of channel is also concentrated in cortical and hippocampal pyramidal cell dendrites, where it plays an important role in determining synaptic input integration and thus neuronal output (2). Therefore, it has been suggested to be involved in physiological processes such as cognition as well as pathophysiological states such as epilepsy (3). Multiple other studies support the HCN role also in the function of sensory neurons and pain sensation, particularly pain associated with nerve or tissue injury (4, 5). Recent evidence suggests that these channels may also be therapeutic targets for treatment of depressive disorders (6).

In the study of Wang *et al.* (2001), the authors investigated two members of the HCN family, HCN1 and HCN2, and generated a series of chimeric channels between these isoforms to determine, through electrophysiological experiments, how different channel regions contribute to HCN gating and cAMP modulation (7). They identified structural domains in HCN1 that are important for the basal voltage dependence of gating and the extent to which cAMP modulates the gating (7). Specifically, channels containing either the core transmembrane domain (TMD) or C-terminus including the cyclic nucleotide-binding domain (CNBD) of HCN2 channels activate at more negative voltages compared with channels that contain the corresponding regions of HCN1. Differences in cAMP modulation are largely localized to the C-terminus, with channels containing the HCN2 C-terminus showing a larger shift in response to cAMP compared to channels containing the HCN1 C-terminus (7). Thus, removal of the entire C-terminus fully eliminates the response to cAMP (8). Yet the precise nature and molecular mechanism of this structural modulation is unknown (for review see Robinson and Siegelbaum (1)).

HCN1 structures in its apo-state and cAMP-bound have already been solved by cryo-EM (9), together with other HCN1 variants (10). Another group reported a full panorama of structures on HCN4, in its apo-state and cAMP-bound, in presence of different detergents. The group also presents a HCN4 structure with the gate open shedding light onto gating movement and ion permeation in HCN4 pacemaker channels (11). Importantly, more recently, cryo-EM structures of HCN1 in closed, open, and putative intermediate states have been reported. This study aimed to elucidate the mechanisms underlying channel gating driven by voltage-sensor-mediated conformational changes. To isolate these effects, cAMP binding was controlled by saturating the cAMP concentration across all experimental conditions, thereby minimizing variability arising from cAMP-dependent modulation (12).

Although multiple HCN structures have been resolved, the full regulation of HCN gating remains incomplete. In particular, how the different domains contribute to channel gating, how they communicate with one another, and how these interactions modulate channel gating remains an open field for further investigation. Because the effects of cAMP binding are isoform-dependent, it is crucial to account for these differences when investigating the structural basis of HCN gating regulation (13).

Our study builds on the HCN1 and HCN2 chimeric constructs previously engineered and functionally characterized in the literature (7, 8) to investigate the structural determinants of the distinct gating properties and cAMP sensitivity of HCN channels. Specifically, we aim to provide structural insight into how the C-terminus influences HCN gating. To investigate its role in cAMP-dependent modulation, we expressed and purified two HCN variants: HCN112, engineered and functionally characterized by Wang *et al.* (7), and HCN1ΔC-term (hereafter HCN1ΔC) first engineered and functionally characterized by Wainger *et al.* (8) and further characterized by Wang *et al.* (7, 8).

HCN112 is a chimera comprising the cytoplasmic N-terminus and TMD of HCN1 fused to the cytoplasmic C-terminus of HCN2. This construct was shown to confer stronger cAMP regulation than that observed in HCN1 (7). On the other hand, HCN1ΔC (originally termed HCN1ΔC-term) has a deletion corresponding to the full cytoplasmic C-terminus including the CNBD and C-linker. These functionally well characterized constructs allowed us to structurally investigate whether the C-terminal domain acts in an auto-inhibitory manner on channel gating (8).

## Results

### Structure determination

In recent years, several research groups have published three-dimensional structures for three of the four HCN isoforms, namely HCN1, HCN3 and HCN4 channels (9, 10, 11, 12, 14). For HCN2, however, only the three-dimensional structure of the isolated CL-CNBD has been elucidated so far (15, 16). To study the C-terminal region of the HCN2 channel not only as an isolated protein but in a full channel environment, we took advantage of the HCN112 chimera (Fig. 1*A*) (7). This chimera is composed of the N-terminal domain and TMD of HCN1 and the C-terminal domain of HCN2 channel (7) (Fig. S1). For the cryo-EM studies, we generated an HCN112 construct incorporating a truncation previously used by Lee and MacKinnon for HCN1 (9) to improve protein stability, as the corresponding region is conserved in HCN2 (Fig. S1*B*, and see Methods).

**Figure 1:**
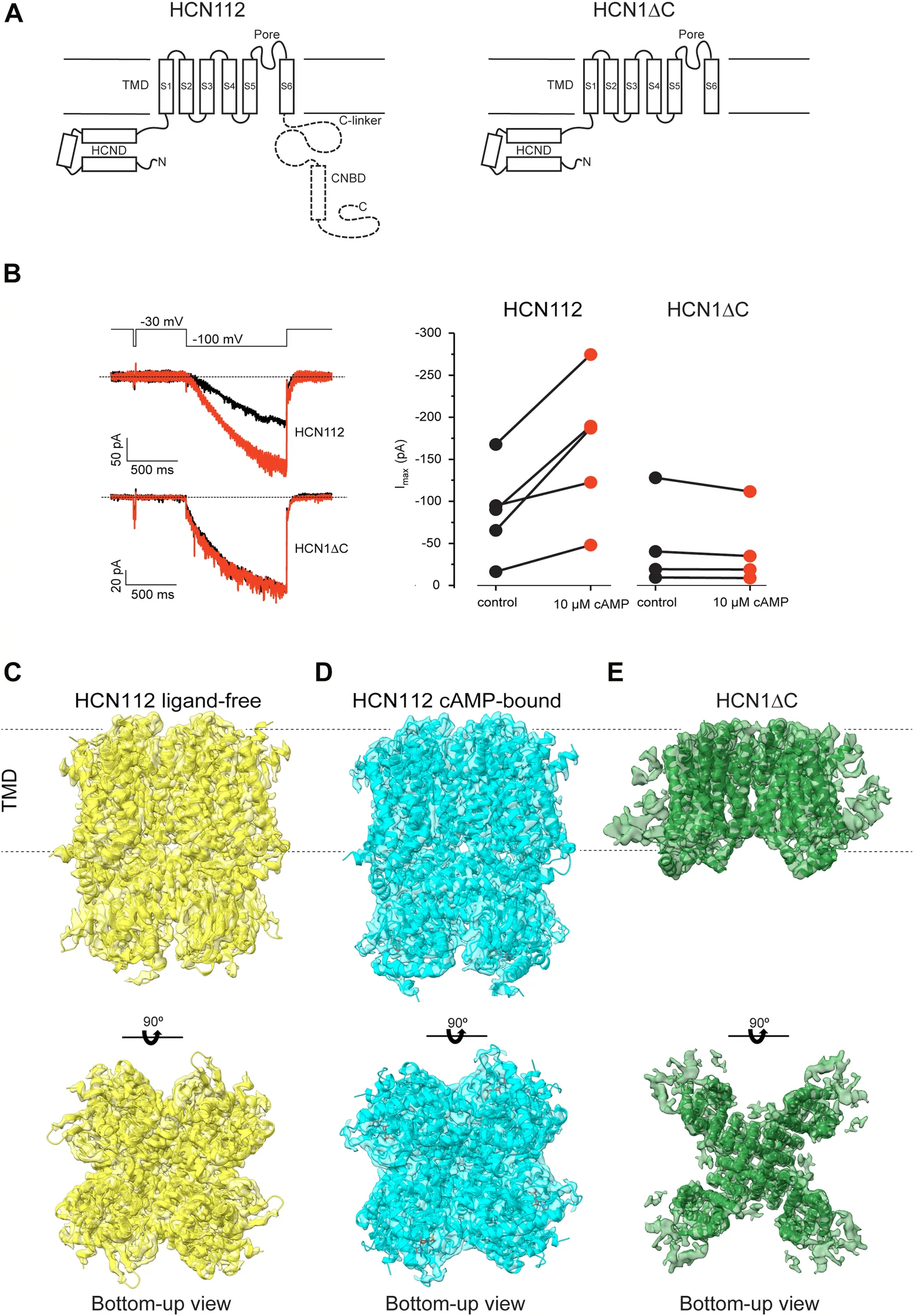
**Structure and function of HCN112 and HCN1ΔC channels.***A,* transmembrane topology of the HCN112 and HCN1ΔC channel constructs. The *dashed line* in HCN112 indicates the C-linker and CNBD, which have been replaced with the corresponding sequence from HCN2 channels. *B,* representative current traces from HCN112 and HCN1ΔC channels expressed in *Xenopus* oocytes and evoked by a voltage step to −100 mV in the absence *(black)* and presence *(red)* of 10 μM cAMP. The graphs on the right show the summary of peak current amplitudes from four individual experiments. *C–E,* cryo-EM maps of HCN112 ligand-free *(yellow)*, HCN112 cAMP-bound *(cyan)* and HCN1ΔC *(green)*. The 3D-structures are docked in the map and shown in cartoon representation. The top panels are side-views, the *bottom panels* are a *bottom-up views*. CNBD, cyclic nucleotide-binding domain; HCN, hyperpolarization-activated, cyclic nucleotide-gated.

First, as a functional control, we assessed the effect of cAMP on the current amplitude in inside-out macropatches from *Xenopus laevis* oocytes expressing HCN112, to confirm that the construct we expressed exhibits the cAMP sensitivity previously reported in the literature (7). Data presented in Figure 1*B* (left panel) show that the ability of cAMP to increase the maximum current amplitude, *I*_max_, evoked by a hyperpolarizing voltage jump is preserved upon binding to the HCN2 CNBD in the HCN1 background. These data confirm that the HCN112 chimera expressed in our system retains the cAMP-dependent functional response previously established for this chimera (7), supporting its use as a valid tool to structurally investigate the role of the CNBD in a functional channel system. The noise observed in some of the current traces may be attributable, at least in part, to the quality of the recordings; however, the recordings were of sufficient quality for the functional control experiments presented here. It is important to note that we did not pursue a detailed electrophysiological characterization of HCN112 channel, since this work was already carried out by Wang and colleagues (7). Because of the truncation introduced for the cryo-EM studies, further functional characterization of this construct could be pursued in future studies. However, since the same truncation has been shown not to affect HCN1 channel function (9), and the corresponding region is conserved in HCN2, we expect that it is unlikely to substantially affect HCN2 channel function.

We therefore expressed and purified HCN112 chimera in the presence (cAMP-bound) or absence (ligand-free) of cAMP, according to Lee and MacKinnon (9). Cryo-EM density maps of HCN112 in the ligand-free and cAMP-bound states were determined to an overall resolution of 3.5 Å and 3.26 Å, respectively (Figs. 1, *C*, *D*, S2–S5, Table S1). This is comparable to the resolution of HCN1 (3.5 Å) by Lee and MacKinnon (9).

In addition, to investigate the structural consequences of complete CNBD removal in the HCN1 variant, we expressed and purified the previously engineered and functionally characterized HCN1ΔC construct (originally termed HCN1ΔC-term) (7, 8), also according to Lee and MacKinnon (9). As for HCN112, a functional control was also performed but no further detailed electrophysiological characterization, as the functional properties of HCN1ΔC have also been previously established (7, 8). The resulting cryo-EM reconstruction for HCN1ΔC reached a global resolution of 3.9 Å (Figs. 1*E*, S6 and S7, Table S1).

Both HCN112 ligand-free and HCN112 cAMP-bound reconstructions exhibited high sphericity (<0.96 out of 1), indicating overall good directional resolution and isotropic particle distribution (Fig. S8, *A* and *B*). Consistently, the 2D class averages for both datasets display a broad range of particle orientations (Figs. S2 and S4). In agreement with this, the raw (unsharpened) maps show well-preserved structural features, supporting the overall quality of the reconstructions (Fig. S9, *A* and *B*).

HCN1ΔC shows lower directional resolution than HCN112 (sphericity 0.852; Fig. S8*C*), consistent with the limited range of particle views observed in 2D class averages (Fig. S6). Noteworthy, this construct proved challenging during data collection and processing and was therefore analyzed with particular attention. Both raw (Fig. S9*C* and S10) and sharp maps were analyzed alongside, and the overall structural impact of the complete CNBD deletion could be investigated.

### C-terminal region of HCN2 shows a compressed conformation in an HCN1 channel background

HCN112 chimera shares the overall architecture of previously described HCN channels. The global channel structure of HCN112 in the ligand-free state is comparable to HCN1 ligand-free (PDB 5U6O) with an RMSD of 1.4308 Å for 1841 residues aligned (Fig. 2*A*). The isolated C-linker + CNBD structure in the cAMP-bound state (PDB code 1Q5O) (15) is also very similar to HCN112 + cAMP with a RMSD of 1.1097 Å for 195 residues aligned. Similar to HCN1, HCN112 channel consists of four identical subunits forming a 4-fold symmetric channel which is surrounded by the membrane. It has a membrane-embedded set of six alpha-helices (S1-S6,TMD constituting the pore (S5-S6) and the voltage sensor (S1-S4). The HCN112 C-terminal cytoplasmic domain (CD) is formed by the C-linker and the CNBD. Unique to HCN channels is the N-terminal HCN domain spanning the first 45 residues preceding S1 and forming a 3-alpha-helical domain (Fig. S1). It has been shown by Porro *et al.* (17) and Wang *et al.* (18) that the HCN domain couples voltage gating and the cAMP response in HCN channels. This domain is wedged between the voltage sensor and the CD. The HCN domain contacts the S4 helix from the same subunit and the C-linker and CNBD from an adjacent subunit. This constrains the channel at the level of the C-linker disk (9).

**Figure 2:**
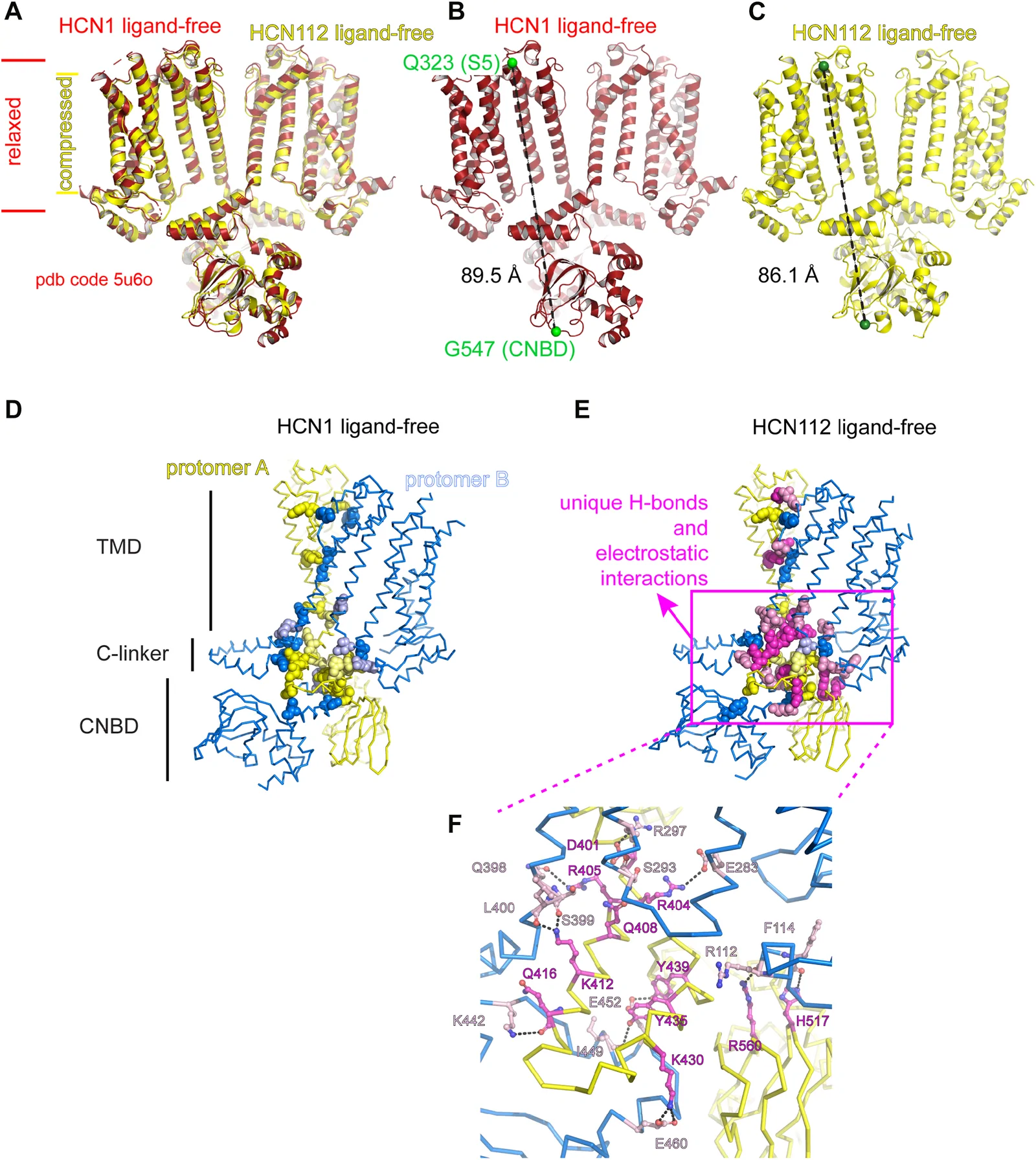
**HCN112 channels show a compressed conformation and unique inter-chain interactions.***A–C,* cartoon representations of HCN1 ligand-free *(B, red, pdb code*5U6O*),* HCN112 ligand-free *(c, yellow)* and overlay *(A)*. Only two opposing subunits are shown for clarity. The *green spheres* and the *dashed line* show the distance between the *top* of S5, residue Q323 and the distal end of the CNBD, residue G547. *D–E,* inter-chain interactions between protomor A *(yellow ribbon)* and protomor B *(blue ribbon)* for HCN1 ligand-free (*D,* pdb code 5U6O) and HCN112 ligand-free *(E)*. Interacting residues are shown in sphere representation. Hydrogen bonds are shown between yellow (protomor A) and *dark blue* (protomor B) residues. Salt bridges are shown between *pale yellow* (promotor A) and *pale blue* (protomor B) residues. Hydrogen bonds and salt bridges that are unique in HCN112 *(E)* are shown as *magenta* and *pink residues*. *F,* detailed view of the uniquely interacting residues in the C-linker region. Residues of protomor A are shown in *magenta ball* and *stick* representation; residues of protomor B are shown in *pink*. Oxygen atoms are shown in *red*, nitrogen atoms are shown in *blue*. CNBD, cyclic nucleotide-binding domain; HCN, hyperpolarization-activated, cyclic nucleotide-gated.

Besides these above-mentioned similarities in the overall channel structure, comparison of HCN112 and HCN1 (PDB 5U6O) in the ligand-free states reveals an important change in the quaternary conformation (Fig. 2). In HCN112, the CD and TMD are closer to each other in the direction perpendicular to the plane of the membrane, suggesting a ‘compressed’ mode of the TMD and CNBD (Supplementary Movie S1) in HCN112 compared to a ‘relaxed’ mode in HCN1. This compression amounts to ∼3.5 Å when measuring the distance from the top of the S5 helix (residue Q323) to the distal end of the CNBD (residue G547) (Fig. 2, *A*–*C*). This compression is also observed in the HCN112 cAMP-bound structure compared with the HCN1 cAMP-bound (PDB 5U6P) (Fig. S11). Importantly, as a result of the compression movement in HCN112 we observe a drastic increase in the inter-chain interactions through hydrogen bonds and salt bridges (Fig. 2, *D*–*F*, Table 1, Figs. S12 and S13). An important limitation of our contact analysis is that minor coordinate shifts can artificially cause a bond to "appear" or "disappear" during the gating process. These interactions (indicated in purple, Fig. 2, *E* and *F*) are unique for HCN112 and not present in HCN1. For example, in the pore helix and middle of S6 there are unique H-bond interactions between chain A residues L357, Y386 and chain B residue C358. Additionally, at the top of the pore helix there is a unique H-bond between chain A residue A363 and chain B residue Q364. However, most of the unique interactions in HCN112 between chain A and B are localized in the C-linker, a region that has been shown repeatedly to be important for coupling ligand binding to channel opening (7, 11, 15, 19, 20, 21, 22, 23, 24, 25, 26, 27, 28, 29, 30, 31, 32, 33, 34, 35, 36, 37, 38) (Fig. S14*A*). In a recent study, the C-linker has also been implicated in the gating polarity of a CNBD channel chimera composed of the EAG pore domain in a HCN1 background (39). The unique inter-chain salt bridges in the C-linker of HCN112 are D401-R297, R404-E283 and K430-E460. The unique inter-chain H-bonds are R405-Q398, Q408-S293, K412-S399/L400, Q416-K442, Y435-I449, Y439-E452 in the C-linker. Lastly, there are unique inter-chain H-bond involving the CNBD, namely residues H517-F114 and R560-R112 (Fig. 2*F*, Figs. S12 and S13). As shown in Table 1 most of the H-bonds present in HCN112 but not in HCN1 are localized in the C-linker, a region important for coupling ligand binding to channel opening (Fig. S14*A*).

**Table 1:** Unique H-bonds and salt bridges observed in hyperpolarization-activated, cyclic nucleotide-gated 112 channel but not in hyperpolarization-activated, cyclic nucleotide-gated 1 channel

Salt bridges	cAMP-free	cAMP-bound
CL	D401-R297	
	R404-E283	
	K430-E460	

**H-bonds**		

TMD	L357-C358	
	A363-Q364	
	Y386-C358	
CL	R405-Q39	
	Q408-S293	
	K412-S399/L400	K412-L400
	Q416-K442	
		H421-N465
		Y434-E452
	Y435-I449	
	Y439-E452	Y439-E452
		V495-N454
		R548-E575
CL- cyclic nucleotide-binding domain	H517-F114	
	R560-R112	

In summary, in HCN112 we observe a more compressed conformation of the structure in the plane perpendicular to the membrane compared to HCN1. As a result, there are more inter-chain interactions, especially in the C-linker which couples the CNBD to the TMD. We suggest that this compressed conformation is one of the reasons why cAMP works more efficiently in HCN2 than in HCN1 channels. An important remark is that the HCNΔC structure may not fully represent a naturally occurring auto-inhibition-relieved state. Nevertheless, a high-resolution structure of full-length HCN2 is still required to comprehensively define the structural determinants underlying its more efficient cAMP-dependent modulation relative to HCN1. The only previously available HCN2 structures are limited to the intracellular domain (isolated C-linker and CNBD) (15) and therefore represent an incomplete channel. Comparison of the HCN2 intracellular domain (PDB 1Q5O) with our HCN112 chimera structure suggests subtle differences at the interface between the transmembrane and intracellular domains. However, these variations may arise from the absence of the transmembrane region of HCN2 (PDB 1Q5O) rather than reflecting intrinsic features of the full-length HCN2 channel.

### cAMP binding breaks interactions in the compressed HCN112 conformation

To study the compressed mode and the unique inter-chain interactions in HCN112 CL-CNBD in more detail, we next analyzed HCN112 in its cAMP-bound form.

The structure of cAMP-bound HCN112 superimposes onto the ligand-free HCN112 structure with an RMSD of 0.542 Å (Fig. 3, *A*–*C*). The largest conformational change upon cAMP-binding can be observed in the CNBD, which rotates and tilts away from the C-linker, and toward the C-terminal helices D and E (Movie S2 and Fig. S13). These helices rigidify and become more structured upon cAMP binding.

**Figure 3:**
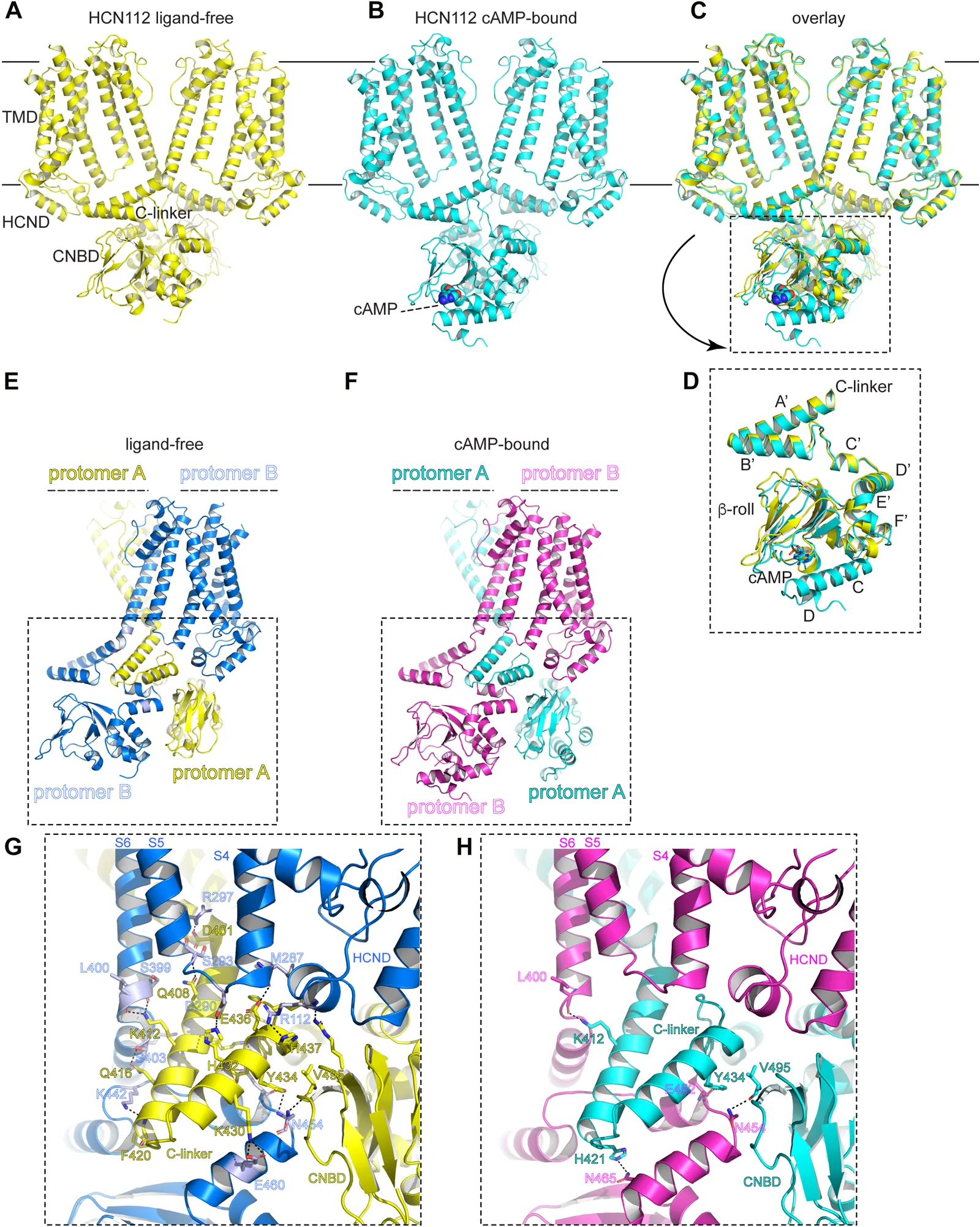
**cAMP binding causes a conformational change of the C-linker and CNBD, and breaks inter-chain interactions in HCN112.***A–C,* cartoon representation of HCN112 in the ligand-free conformation *(A, yellow)*, cAMP-bound conformation *(B, cyan)* and overlay *(C)*. Only two opposing subunits are shown for clarity. *D,* the inset shows a detailed view of conformational changes in the C-linker and CNBD. *E–H,* interchain-interactions between promotor A *(yellow)* and promotor B *(blue)* in the HCN112 ligand-free conformation and protomer A *(cyan)* and protomer B *(magenta)* in the HCN112 cAMP-bound conformation. *G and H,* the insets show a detailed view of inter-chain interactions. Interacting residues are shown in ball and stick representation. *Dashed lines* show hydrogen bonds or salt bridges. Oxygen atoms are shown in red, nitrogen atoms are shown in *blue*. CNBD, cyclic nucleotide-binding domain; HCN, hyperpolarization-activated, cyclic nucleotide-gated.

As a result of this conformational change there is a large decrease in the number of inter-chain interactions under the form of hydrogen bonds and salt bridges. Specifically, there are 17 inter-chain H-bonds and salt bridges in the ligand-free form of HCN112 (see Table 1). Most of these interactions are broken in the cAMP-bound state except for three remaining H-bonds (K412-L400, Y434-E452, Y439-E452) and 3 H-bonds that are newly formed (H421-N465, V495-N454, R548-E575) (Figs. 3, *F*–*H*, S15 and S16). For HCN112 in the ligand-free conformation we will only discuss those interactions that likely contribute to gating of the channel.

First, a key role appears to be played by R112 in the HCND domain. Three residues project interactions onto R112, namely H437, Q440 (in the C-linker) and R560 (in the CNBD). Second, there are four residues at the bottom of S4 and the S4-S5 linker that interact with the C-linker. These include M287 (S4), D290 and S293 (S4-S5 linker), and R297 (S5). Lastly, two residues at the bottom of S6, namely S399 and L400 interact with K412 in the C-linker (Fig. 3*G*).

In summary, our interpretation is that there are tight inter-chain interactions in the CNBD and C-linker, also involving the HCND domain and S4-S5 linker, and that these interactions are broken upon cAMP-binding. The breaking of these interactions could then contribute to the facilitation of channel opening. Although our structural data identify unique contacts and a compressed conformation, they do not establish a causal link to the enhanced cAMP sensitivity of HCN112. Functional studies will be required to test this proposed mechanism.

### Deletion of the C-linker and CNBD causes partial channel opening in the HCN1ΔC channel

It has been shown that the intracellular channel region is not only responsible for mediating cAMP regulation, but also for the integrity of the channel gate, which is formed by the intracellular helix bundle of the S6 segments (8). Previous studies proposed a gating mechanism in which the C-linker disk plays an important role in keeping the gate closed at depolarized voltages: 1) by forming intense contacts with the unusual S4 helix (9), and 2) by contacting the HCN domain of opposite subunits (37). These interactions help to stabilize the gate in a closed right-handed helix bundle. For channel opening, the CL-CNBD has to perform a leftward rotation to unwind the right-handed S6 helix bundle gate. Such a leftward rotation is suggested to be induced by the movement of the voltage sensor upon a hyperpolarizing voltage jump. Cyclic nucleotide binding can cause a CL-CNBD leftward rotation, too. However, this cAMP-triggered rotation is not sufficient to open the channel but is supportive for voltage-induced rotation. In this sense, cAMP causes the CL-CNBD to adopt a conformation, which can be interpreted as relief of autoinhibition (8). To test this proposed mechanism, we took advantage of the HCN1ΔC construct, in which the C-terminal CD was deleted (8). This construct was characterized in detail using electrophysiological studies by Wainger *et al.* (8). The deletion spans two domains, the C-linker and the CNBD, starting from D401 until the last residue of HCN1 (Fig. 1, *A*, *B* and *E*). From the mechanism described above we expect that the gate and the pore move towards their open configurations when the CL-CNBD is missing. Gating of the HCN channel is activated by membrane hyperpolarization and is also modulated by cAMP. It has been shown that the CNBD has an inhibitory effect on channel gating that is relieved by cAMP binding (8). Such a relieving effect is reflected by an increase in current amplitude at a given voltage, by a shift of the voltage of half-maximum activation, *V*_1/2_, to more depolarized values, and by an acceleration of activation kinetics and a deceleration of deactivation kinetics (40).

Here, we determined the cryo-EM structure of HCN1ΔC to assess the structural consequences of the absence of the intracellular domain. HCN1ΔC shows an RMSD value of 1.114 Å and 1.096 Å when aligned to the TMD domain of HCN112 ligand-free and cAMP-bound structures, respectively. To evaluate the implications of the intracellular domain deletion in HCN1ΔC, we next performed a pore analysis that included the HCN112 in both ligand-free and cAMP-bound states for comparison. The most pronounced differences between HCN1ΔC and HCN112 are observed in the cavity and gate regions. For instance, at the gate residue Q398, the pore radius in HCN112 ligand-free is 1 Å, whereas in HCN1ΔC it increases to ∼2.5 Å (Figs. 4, *A*, *B*, S17).

**Figure 4:**
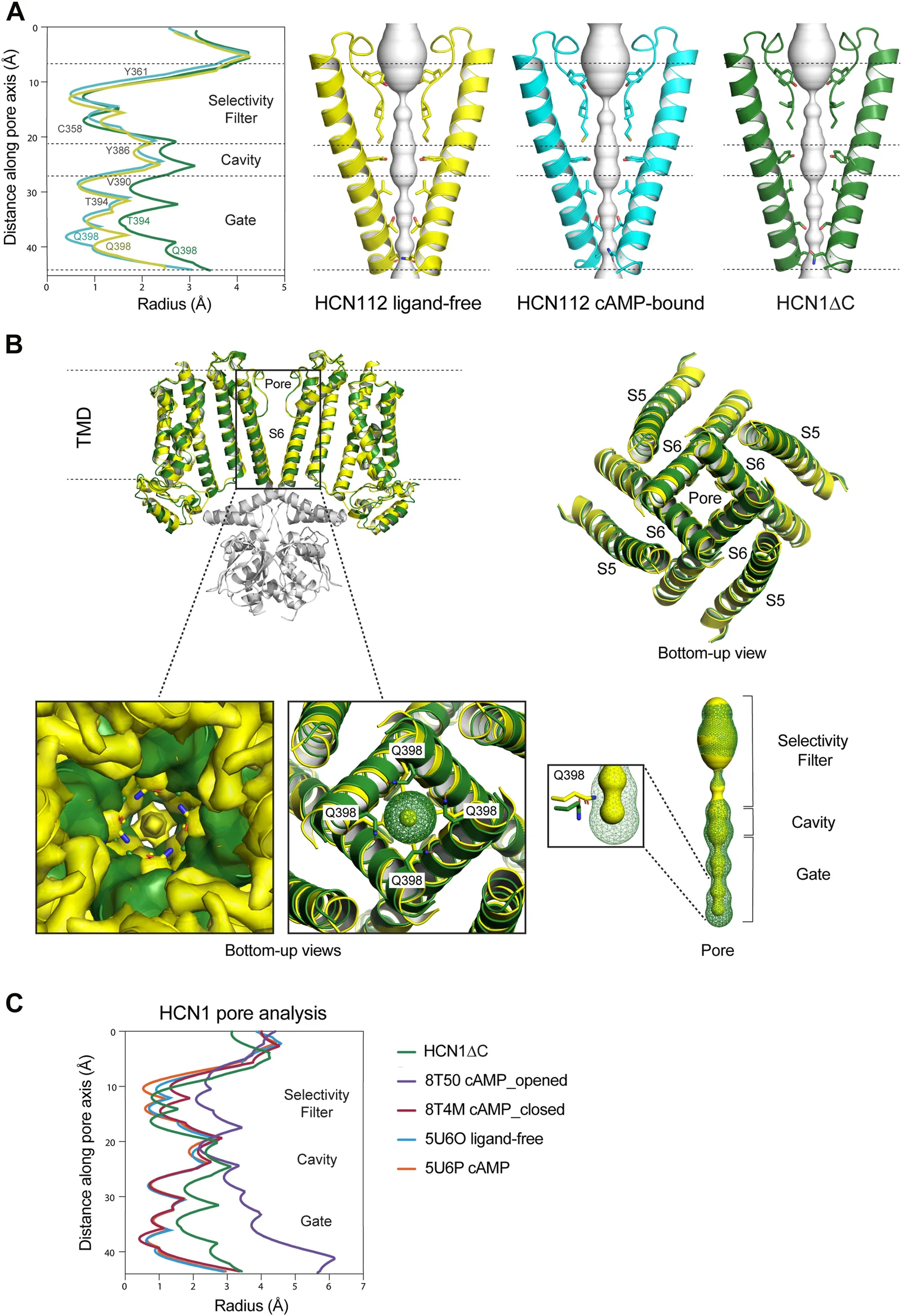
**Relief from auto-inhibition in HCN1ΔC reveals a pore in an intermediately open conformation.***A,* pore radius calculations for HCN112 ligand-free *(yellow)*, HCN112 cAMP-bound *(cyan)*, and HCN1ΔC *(green)* are shown on the graph *(left)*. The corresponding structures of the pore region (*A and C chains,* residues 358–401) are shown as cartoons *(right)*, with the sidechains of key pore-lining amino acids shown as licorice sticks. The selectivity filter, cavity, and gate regions are indicated on both the graph *(left)* and structures *(right)* with *dotted lines*. *Gray spheres* within the structures represent the calculated pore. *B,* overlay of two subunits of HCN112 ligand-free *(yellow)* and HCN1ΔC *(green)*, is shown as cartoons on the *top left*, with a *zoomed bottom-up view* on the *top right*. The C-linker and CNBD domain from the HCN112 ligand-free structure are colored *gray*. A *zoomed-in view* of the pore region in the overlapped structures is displayed in the *bottom panels*. The *bottom left panel* shows the overlapped structures with their corresponding cryo-EM maps, while the *bottom right panel* focuses on displaying the calculated pore radius within the structures *(yellow sphere and green mesh)* and highlighting the residue Q398 for all S6 helices. A side view of the calculated pore for both structures is included in the *bottom-right corner*, emphasizing the position of Q398 in the gate region. *C,* pore radius calculations of the HCN1ΔC structure, compared to published HCN1 structures in different conformational states, are plotted. The approximate locations of the selectivity filter, cavity, and gate regions are indicated for reference. Comparison of the pore radius in the closed ligand-free HCN1 conformation (pdb code 5U6O), the closed cAMP-bound HCN1 conformation (pdb code 5U6P), the closed HCN1 F186C S264C bound to cAMP (pdb code 8T4M), the open HCN1 F186C S264C bound to cAMP (pdb code 8T50) and the intermediately open HCN1ΔC. CNBD, cyclic nucleotide-binding domain; HCN, hyperpolarization-activated, cyclic nucleotide-gated.

Structural analysis of HCN1ΔC reveals a movement of pore helices S6 and S5, as shown in the inset in Figure 4*B* and Movie S3. Overlay of cryo-EM densities of HCN112 ligand-free and HCN1ΔC (bottom left inset) clearly shows this redistribution of helices and inner sidechains along the pore. Notably, the TMD of HCN1ΔC shows limited resolution, making detailed side-chain interpretation difficult and warranting careful consideration when analyzing this region (Fig. S17).

Determinant aspects confirming the widening of the pore in HCN1ΔC are sidechains of V390, T394 and Q398. Interestingly, these residues have also been shown before to be key elements in the inner channel gate (9). The long sidechain of Q398 at the intracellular gate of the pore tilts away from the inner pore cavity, widening the diameter of the intracellular gate of HCN1ΔC (colored in green mesh - bottom right inlet) (Fig. 4). The HCN1ΔC pore radius is close to double in size of the HCN112 pore (Fig. 4*A*), which is depicted in yellow color and overlayed in the bottom-up view (bottom panels) in Figure 4*B*. These small differences in conformation of S6-S5 helices in HCN1ΔC led the overall pore diameter to be halfway toward being fully open (∼3 Å *versus* ∼5 Å) and thus resemble the open HCN1 conformation (Fig. 4*C*).

In summary, we hypothesized that the S6-S5 and S4 helices movements cause a loss of contacts between these pore helices and the C-linker, as well as between the HCND and the CNBD. We therefore suggest that these rearrangements reflect the transition state towards an open pore in HCN1, triggering a release of constraints and allowing the pore to open. We also propose that our HCN1ΔC structure, with its deletion reflecting an absence of auto-inhibition, mimics a transitioning-hyperpolarized state, that most likely precedes the hyperpolarized state described in Lee and MacKinnon (10) (Fig. 5).

**Figure 5:**
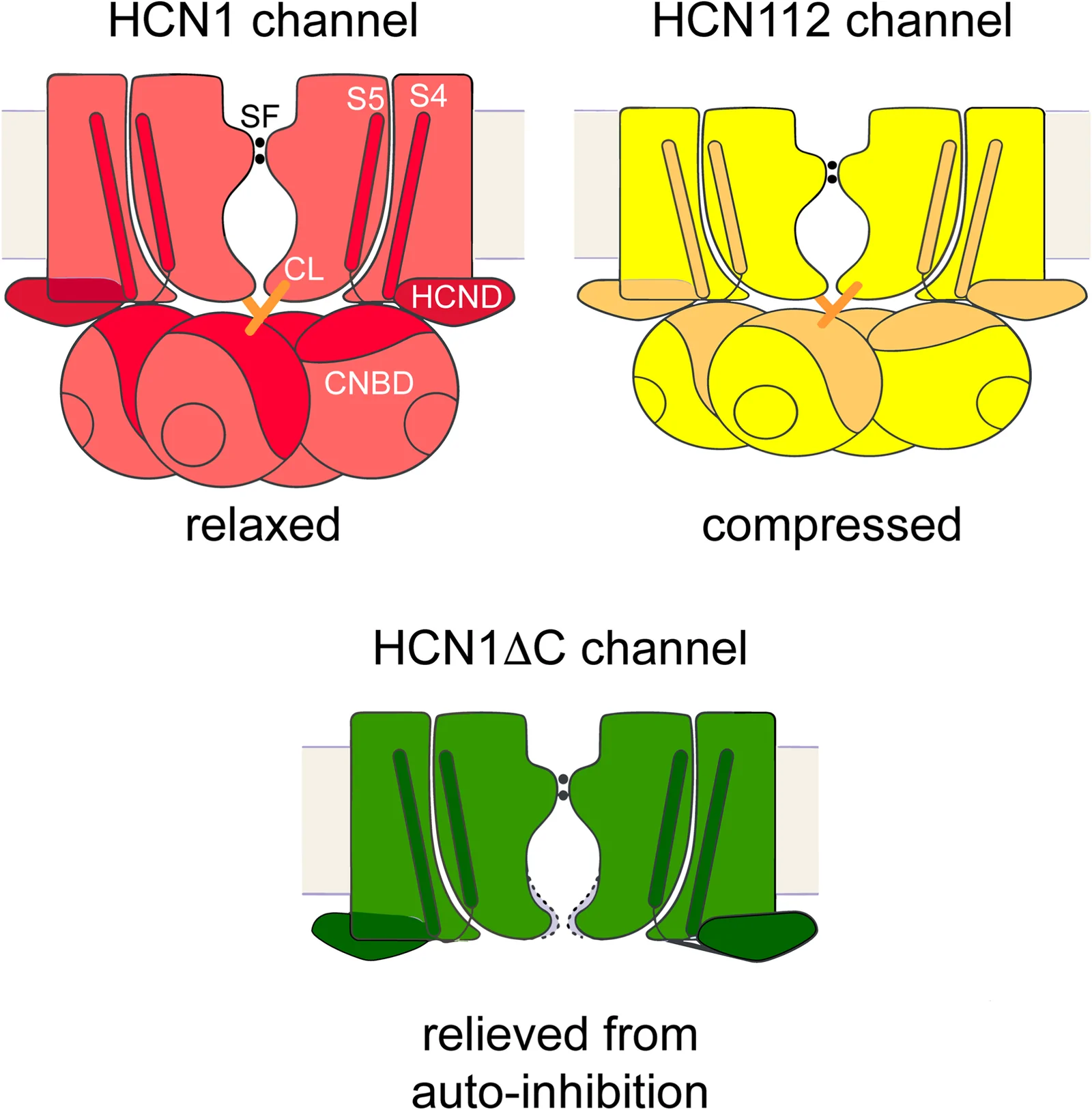
**Models of domain compression in HCN112 and relief from auto-inhibition in HCN1ΔC.** The models illustrate the domain compression in HCN112 *(yellow)* upon comparison with HCN1 in the closed ligand-free conformation *(red*). The *green model* shows relief from auto-inhibition in HCN1ΔC resulting in a partially open pore conformation. The figure was adapted from Lee and MacKinnon (2017) with permission from Elsevier. HCN, hyperpolarization-activated, cyclic nucleotide-gated.

## Experimental procedures

### Constructs design

All HCN1 constructs were engineered in the background of the human HCN1_EM_ plasmid made by Lee and MacKinnon (9) and was cloned into a pEG-BacMam vector (41) for expression in mammalian cells and which tags the protein of interest with both N-His10, eGFP and 3C protease site. The following constructs were generated on top of the HCN1_EM_ constructs: 1. Similar to the HCN112 chimera originally engineered by Wang *et al.* (7), our chimera HCN112 contains region Met1-Leu400 from human HCN1 and Asp401-Leu658 from human HCN2, with the region between Ala705-Ser866 of HCN2 which was deleted, while the HCN2 extreme C-terminal region including the SNL tripeptide sequence was preserved Ala867-Leu889 (residue numbering according to full-length human HCN2, Fig. S1*B*); 2. HCN1ΔC corresponds to the HCN1 gene covering the sequence between Met1-Asp401, with a deletion that spans the entire C-terminal region.

### Electrophysiological recordings

For expression in *Xenopus* oocytes HCN112 and HCN1ΔC were subcloned into pGEM-HE using *Xba*I and *Hind*III restriction sites. Plasmids were linearized with *Nhe*I and transcribed with T7 RNA polymerase using the mMessage mMachine T7 transcription kit. The RNA quality was verified by loading RNA samples on 1% agarose gel.

Oocytes were surgically removed from adult female South African claw frogs *X. laevis* under anesthesia with 0.3% tricaine methanesulfonate (MS-222) (Pharmaq Ltd.). After removal, the oocytes were treated with collagenase A (3 mg/ml; Roche) for 105 min in Ca^2+^-free Barth’s solution containing (in mM) 82.5 NaCl, 2 KCl, 1 MgCl_2_, and 5 Hepes, pH 7.5. Oocytes of stages IV and V were manually dissected and injected with cRNA encoding either HCN112 or HCN1ΔC channels. After injection with cRNA, the oocytes were incubated at 18 °C for 2 to 6 days in Barth’s solution containing (in mM) 84 NaCl, 1 KCl, 2.4 NaHCO_3_, 0.82 MgSO4, 0.41 CaCl_2_, 0.33 Ca(NO3)_2_, 7.5 TRIS, pH 7.4. Oocytes harvested in our lab were complemented with ready-to-use oocytes purchased from Ecocyte Bioscience. The surgery procedures were carried out in accordance with the German Animal Welfare Act with the approval of the Thuringian State Office for Consumer Protection on 30.08.2013 and 09.05.2018.

Macroscopic currents were recorded using the inside-out configuration of the patch-clamp technique. All measurements were started after a delay of 3.5 min to minimize run-down phenomena. Patch pipettes were pulled from quartz tubings with outer and inner diameters of 1.0 and 0.7 mm (VITROCOM), respectively, using a laser puller (P-2000, Sutter Instrument). The pipette resistance was 1.2 to 2.1 MOhm. The bath solution contained (in mM) 100 KCl, 10 EGTA, and 10 Hepes, pH 7.2, and the pipette solution contained (in mM) 120 KCl, 10 Hepes, and 1.0 CaCl_2_, pH 7.2. For parts of the experiments, a saturating concentration of 10 μM cAMP (BIOLOG LSI GmbH & Co. KG) was applied with the bath solution. A HEKA EPC 10 USB amplifier (Harvard Apparatus) was used for current recording. Pulsing and data recording were controlled by the Patchmaster software (Harvard Apparatus). The sampling rate was 5 kHz. The holding potential was generally −30 mV. Maximally two membrane patches were excised from one individual oocyte.

HCN channels have traditionally been regarded as non-selective monovalent cation channels, with Ih/If carried predominantly by Na^+^ and K^+^. Recent single-channel work, however, shows that Ca^2+^ can permeate through HCN2/HCN4 and native Ih/If channels at physiological extracellular Ca^2+^ concentrations. This implies that extracellular Ca^2+^ directly competes with Na^+^ and K^+^ for occupancy of the HCN selectivity filter, such that changes in [Ca^2+^]_o_ can reduce monovalent conductance and alter the mixed-cation reversal potential without necessarily changing channel gating. In our experiments, any Ca^2+^-dependent changes in current amplitude or apparent selectivity therefore likely reflect a combination of Ca^2+^ permeation and competitive occupancy effects in the pore, rather than a simple loss of recording quality or secondary effects alone (42, 43, 44).

### Protein expression, purification and sample preparation for Cryo-EM

The different HCN constructs were expressed in HEK293S GnTI^−^ cells (Goehring *et al.*, 2014) at 37 °C and to a density of ∼3 × 10^6^ cells/ml for ∼72 h, after addition of baculoviruses carrying HCN1 genes (10% v/v). After 15 h, 10 mM sodium butyrate was added to cells and the culture temperature was shifted to 30 °C for ∼72 h, after which cells were harvested and pellets were flash frozen and stored at −80 °C.

Cell pellets were resuspended in 30% glycerol for 10 min and lysed by gentle homogenization in 10 mM Tris pH 8.0, 20 mM KCl, 2 mM DTT, 0.1 mg/ml DNAse, 0.5 mM MgCl_2_, 2 μg/ml leupeptin, 10 μg/ml aprotinin, 1 μg/ml pepstatin, 50 μg/ml benzamidine HCl, 1 mM 4-(2-aminoethyl)benzenesulfonyl fluoride hydrochloride (AEBSF), 30% glycerol for 25 min at 4 °C. The lysate was spun at 39,800*g* for 35 min at 4 °C to sediment crude membranes. The membrane pellet was homogenized and solubilized in the extraction buffer (20 mM Tris pH 8.0, 300 mM KCl, 2 mM DTT, 2 μg/ml leupetin, 10 μg/ml aprotinin, 1 μg/ml pepstatin, 50 μg/ml benzamidine HCl, 1 mM AEBSF, 10 mM LMNG + 2 mM CHS) for 2 h. Solubilized membranes were clarified by centrifugation at 39,800*g* for 35 min, 4 °C. The supernatant was incubated with the GFP nanobody-coupled sepharose resin prepared in-house (45) for 1.5 h at 4 °C and subsequently washed for 10 column volumes with wash buffer (20 mM Tris pH 8.0, 300 mM KCl, 2 mM DTT, 2 μg/ml leupetin, 10 μg/ml aprotinin, 1 μg/ml pepstatin, 50 μg/ml benzamidine HCl, 1 mM AEBSF, 0.05% digitonin). An extra five column volumes of buffer with 1 mM EDTA was used to incubate the proteins of interest overnight at 4 °C with PreScission protease to cleave the GFP tag. HCN variants were eluted straight on a Glutathione Sepharose 4B column and washed out with wash buffer. The eluted proteins were concentrated and purified further through a Superose 6 10/300 column using gel filtration buffer (20 mM Tris pH 8.0, 150 mM KCl, 2 mM DTT, 2 μg/ml leupeptin, 10 μg/ml aprotinin, 1 μg/ml pepstatin, 50 μg/ml benzamidine HCl, 1 mM AEBSF, 0.05% digitonin). Peak fractions of interest were pooled, concentrated to 3 to 4 mg/ml, flash frozen in liquid nitrogen and stored at −80 C.

Once thawed, the purified proteins were spun at 13,000 rpm for 5 min at 4 °C and the supernatant was used for grid preparation. 0.4 mM cAMP was added to 3.5 mg/ml HCN112 in order to perform single particle analysis on the HCN112 cAMP-bound sample. 3 μl of the purified HCN variants were applied on glow discharged AuFoil grid R2/2200 mesh within a Vitrobot IV (FEI) operated a 4 °C, 100% humidity (blot time 3 s, waiting time 3 s) and plunged into liquid ethane.

### Cryo-EM data acquisition

Two data acquisitions were acquired using two Titan Krios transmission electron microscopes (FEI) operated at 300 keV with energy filter, equipped with Falcon four electron detector (ABSL) at a nominal magnification of 130k, with a pixel size of 0.91 Å (HCN112+cAMP and HCN1ΔC). Two other datasets, later merged as one single dataset, were acquired at a nominal magnification of 96k, with a pixel size of 0.81 Å (ligand-free HCN112).

The nominal defocus range chosen was between the following values and changing in increments of 0.2: for apoHCN112 this was 1.6 - to 3.2 μm, for HCN112+cAMP this was 1.2 to 3 μm, for HCN1ΔC this was 1.6 to 3.4 μm. Respectively, the total doses were: 41.42 e^−^/Å^2^ (merged ligand-free HCN112), 32.44 e^−^/Å^2^ (HCN112+cAMP) and 39.81 e^−^/Å^2^ (HCN1ΔC). The exposure time for each image was 6.26 s (ligand-free HCN112), 3.98s (HCN112+cAMP), 6.49s (HCN1ΔC), fractionated over 54, 41, 40 EER fractions, respectively. Acquisition parameters are summarized in Table S1.

### Image processing and single-particle analysis

Movies were corrected for beam-induced motion using MotionCor (relion3.1.1) (46) and contrast transfer function parameters were estimated from motion-corrected images using CTFFIND 4.1. For all datasets, particle picking was performed in crYOLO v1.61_GPU (47) using an integrated general model and cutoff 0.1 and JANNI denoised. Particles were extracted in Relion with a box size of 350px. The particle.star file was then imported in cryoSPARC v.3.2.0 (48), where 2D classification, *ab initio* reconstruction and refinements were performed (Figs. S2–S7 for Cryo-EM flowchart). During refinement, C4 symmetry was imposed in all datasets. The same single-particle processing was applied for all datasets, leading to a local resolution of 2.9 Å ligand-free HCN112, 2.7 Å HCN112+cAMP, 3.45 Å HCN1ΔC.

### Model building, refinement and validation

Manual building and real-space refinement was performed using HCN1 (PDB 5U6O) as starting model for the transmembrane region (TM) and HCN2 crystal structure (PDB 3U10) for the CD in Coot v0.8.9.2 (49) and then in Phenix v1.19.2 (50). These homology models were docked into the density map of the ligand-free HCN112 using UCSF chimera (51) in order to build a starting model for one protomer of the chimeric HCN112. A tetramer model of the channel was obtained by fitting copies of the same model in the C4 symmetric map of the tetramer. The starting model for the cAMP-bound HCN112 was the refined ligand-free HCN112. The placement of the cAMP ligand in the holoHCN112 was based on the crystal structure of the HCN2 in complex with cAMP (PDB 3U10). As a model for HCN1ΔC TM region, the cAMP-bound HCN112 was used but manual rebuilding was necessary for amino acid fragments that undergo conformational changes *i.e.* S6, S5 and HCN domain.

The final ligand-free structure includes residues 97 to 240, and 252 to 579, whereas the cAMP-bound structure includes additional residues 580 to 605 and part of the E-helices built as a poly-Ala helix. For the HCN1ΔC structure building was more challenging due to the lower quality of the cryo-EM map, but residues of the pore region (361–398) could be built using the HCN112+cAMP structure as a reference thus allowing analysis of the pore radius profile. The quality of the final models was evaluated by MolProbity (52) and EMRinger (53) (Table S1). Figures were prepared using Chimera (51), ChimeraX (54) and PyMOL Molecular Graphics System (v1.8, Schrodinger, LLC). Pore analysis was performed in HOLE v2.2.005 (55) after superposition of all protein structures of interest. Interactions between residues were calculated using Contact/Act in the CCP4 suite (56) using a maximum distance cut-off of 3.8 Å. H-bonds and salt bridges were considered between 2.6 to 3.4 Å and van der Waals interactions were considered between 3.2 to 3.8 Å.

## Data availability

Cryo-EM density maps of HCN112 apo, HCN112+cAMP, and HCN1ΔC have been deposited in the electron microscopy data bank under accession code EMD-53516, EMD-53514 and EMD-53515, respectively. Atomic coordinates of HCN112 apo, HCN112+cAMP and HCN1ΔC have been deposited in the protein data bank under accession code 9R1V, 9R1T and 9R1U, respectively.

## Supporting information

This article contains supporting information.

## Conflict of interest

The authors declare that they have no conflicts of interest with the contents of this article.
