# Supplementary material for: Structural basis of isoform-specific cAMP regulation and autoinhibition in hyperpolarization-activated cyclic nucleotide-gated channels
Source: J Biol Chem. 2026 Sep 23;302(11):113589. doi: 10.1016/j.jbc.2026.113589 (PMC13715422; doi:10.1016/j.jbc.2026.113589)
Supplement: Supplementary file 1 [file mmc1.pdf]

Structural basis of isoform-specific cAMP regulation and autoinhibition in hyperpolarization-activated cyclic nucleotide-gated channels

Zorzini V.[1*], Kleiz-Ferreira J.[1], Brams M.[1], Gallardo R.[2*], De Gieter S.[3,4], Kusch J.[5], Ulens C.[1]

[1]Laboratory of Structural Neurobiology, Department of Cellular and Molecular Medicine, Faculty of Medicine, KU Leuven, 3000 Leuven, Belgium.
[2]School of Molecular and Cellular Biology, Faculty of Biological Sciences and Astbury Centre for Structural Molecular Biology, University of Leeds, Leeds, LS2 9JT, United Kingdom.
[*]Current affiliation: Switch Laboratory, Department of Cellular and Molecular Medicine, KU Leuven, 3000 Leuven, Belgium.
[3]Center for Structural Biology, Vlaams Instituut voor Biotechnologie, 1050 Brussels, Belgium.
[4]Structural Biology Brussels, Department of Bioengineering Sciences, Vrije Universiteit Brussel, 1050 Brussels, Belgium.
[5]Institut für Physiologie II, Universitätsklinikum Jena, Jena, Germany.

Correspondence to: chris.ulens@kuleuven.be

Supplementary Fig. 1

A

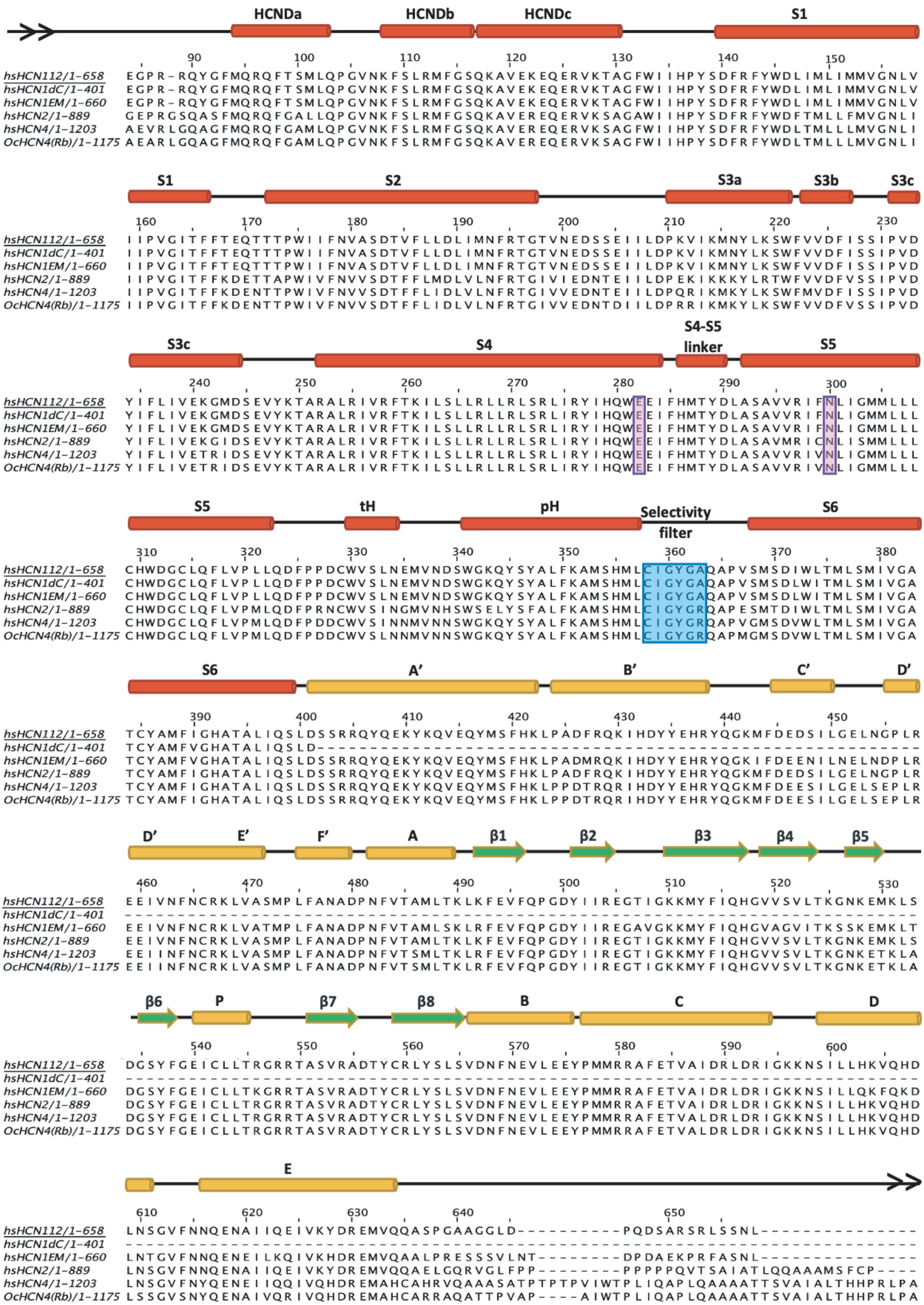

Supplementary Fig. 1

B

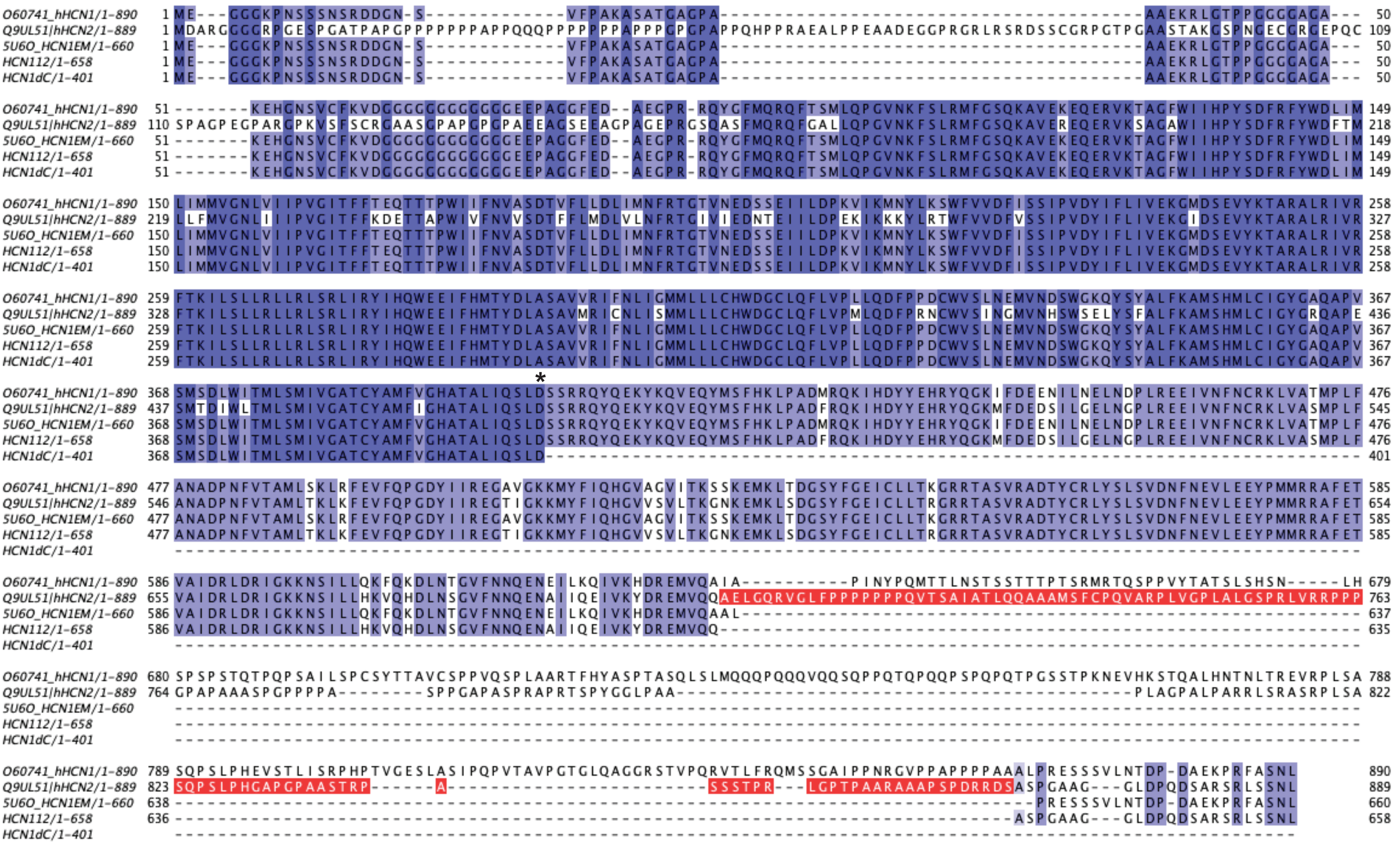

Supplementary Fig. 1 | Sequence alignment and secondary structure elements of HCN channel variants. A) Human sequence of HCN1 EM construct, HCN112 and HCN1ΔC are shown and aligned between them and to human HCN2 (Uniprot code: Q9UL51) and HCN4 sequences (Uniprot code: Q9Y3Q4). In addition, rabbit HCN4 sequence is also represented and aligned (Uniprot code: Q9TV66). Secondary structure elements are denoted by cylinders (-helices, red), arrows (-strand, green) and lines (loops, black). Selectivity filter residues are highlighted with a cyan box. Cytoplasmic domain (C-linker and CNBD) is shown in yellow. Residues that are forming a polar contact between S4 (E403) and S5 (N421) in the closed pore conformation are highlighted with a magenta box. This polar contact is absent in the open pore conformation in HCN1ΔC. N- and C-terminus have been omitted for all the sequences shown. B) Sequence alignment of human HCN1 (Uniprot accession number O60741), human HCN2 (Uniprot accession number Q9UL51), the $HCN1_{EM}$ construct for cryo-EM (PDB code 5u6o), the HCN112 chimera and the HCN1ΔC truncation. Residues are colored in shades of blue according to sequence identity with a cut-off value of 80%. The splice site for HCN112 is Asp401 as indicated with an asterisk. This site is also the truncation point for HCN1ΔC. In HCN112, the residues from Ala705-Ser866 of HCN2 were deleted (indicated in red), while the HCN2 extreme C-terminal region including the SNL tripeptide sequence was preserved Ala867-Leu889 (residue numbering according to full-length human HCN2).

Supplementary Fig. 2

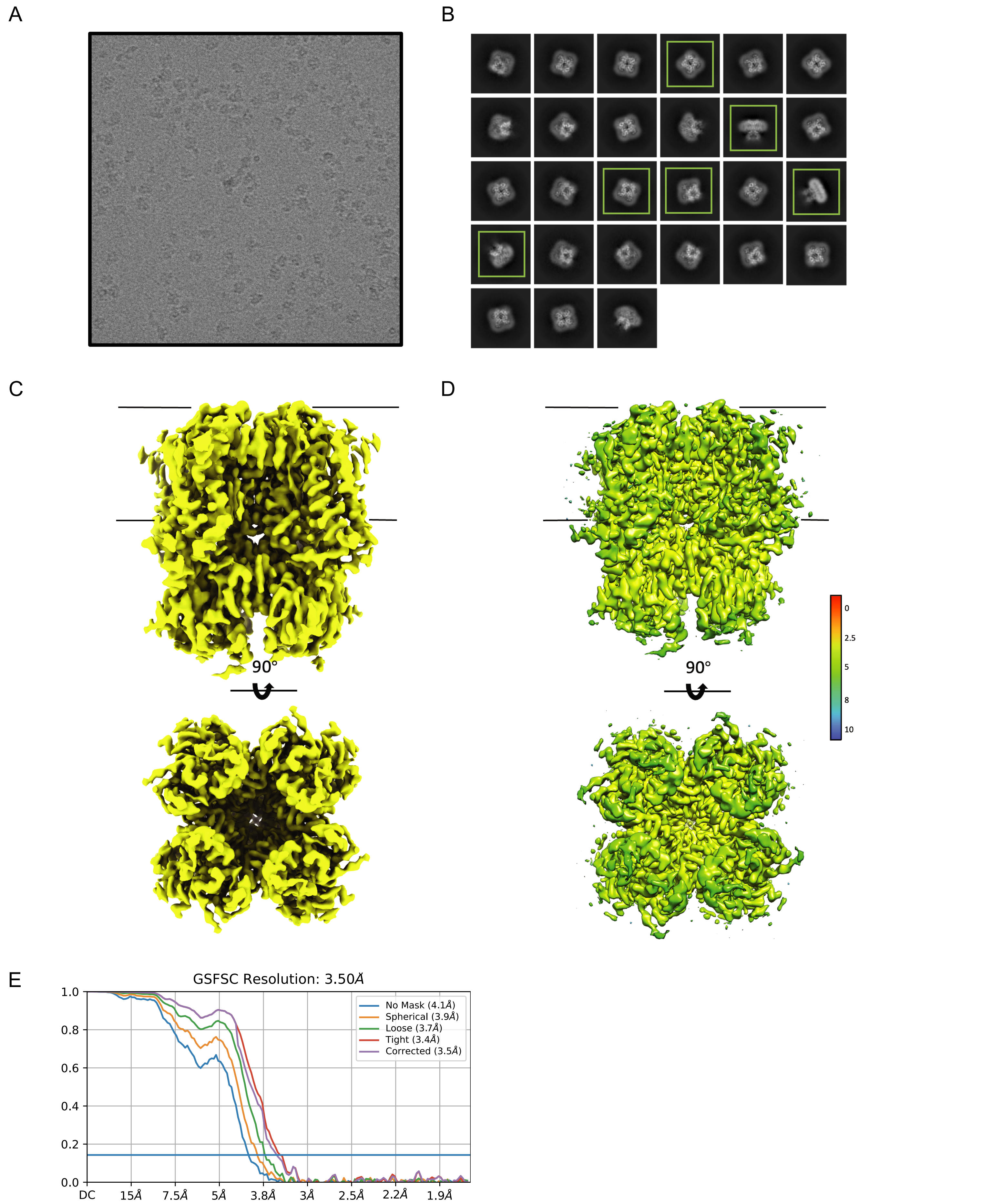

Supplementary Fig. 2 | Single-Particle Cryo-EM of HCN112, ligand-free state. A) Representative raw micrograph of the HCN112 in its ligand-free state. B) Selected 2D class averages. Examples of bottom, tilted, and side views are highlighted with green squares. C) HCN112 density map sharpened with a B-factor of -177.4 Å2. D) Local resolution of the density map estimated using Cryosparc_v.0.3 varying between 3.53-2.9 Å and colored with Chimera software. E) Fourier shell correlation (FSC) plot of HCN112 ligand-free.

Supplementary Fig. 3

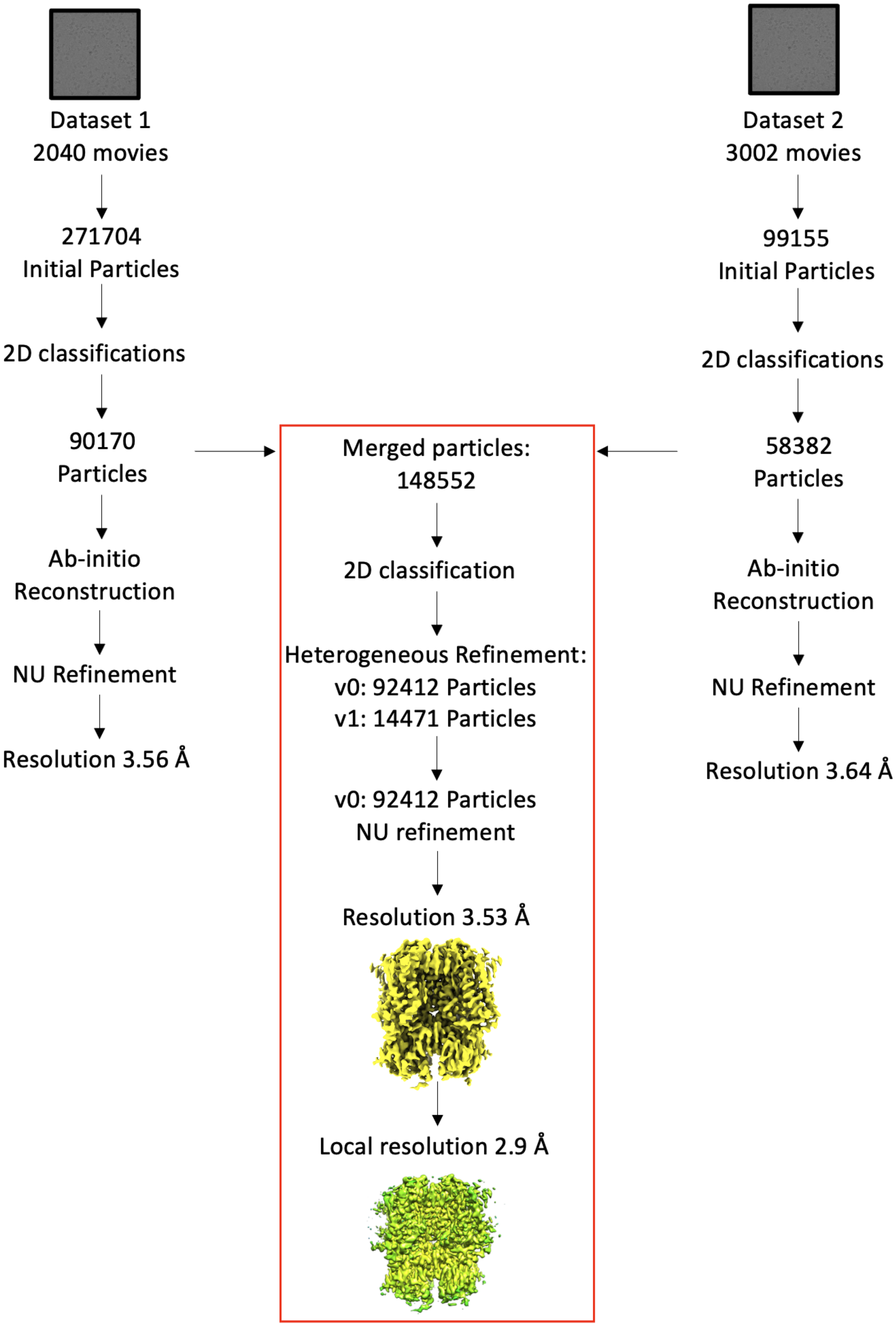

Supplementary Fig. 3 | Single-Particle cryo-EM processing workflow of HCN112, ligand-free state. Processing summary for HCN112 ligand-free state. Initial processing steps that include initial number of particles used and multiple rounds of 2D classification were done in parallel, independently, on the two datasets, as well as the following steps in processing as ab-initio and refinement steps. However, the 2 datasets were merged and duplicated particles were removed before extra steps of 2D classification. NU refinement was performed only on one volume as output of a heterogeneous refinement. A resolution of 3.53 Å was achieved with 92412 particles. Local resolution of density map shown reached 2.9 Å.

Supplementary Fig. 4

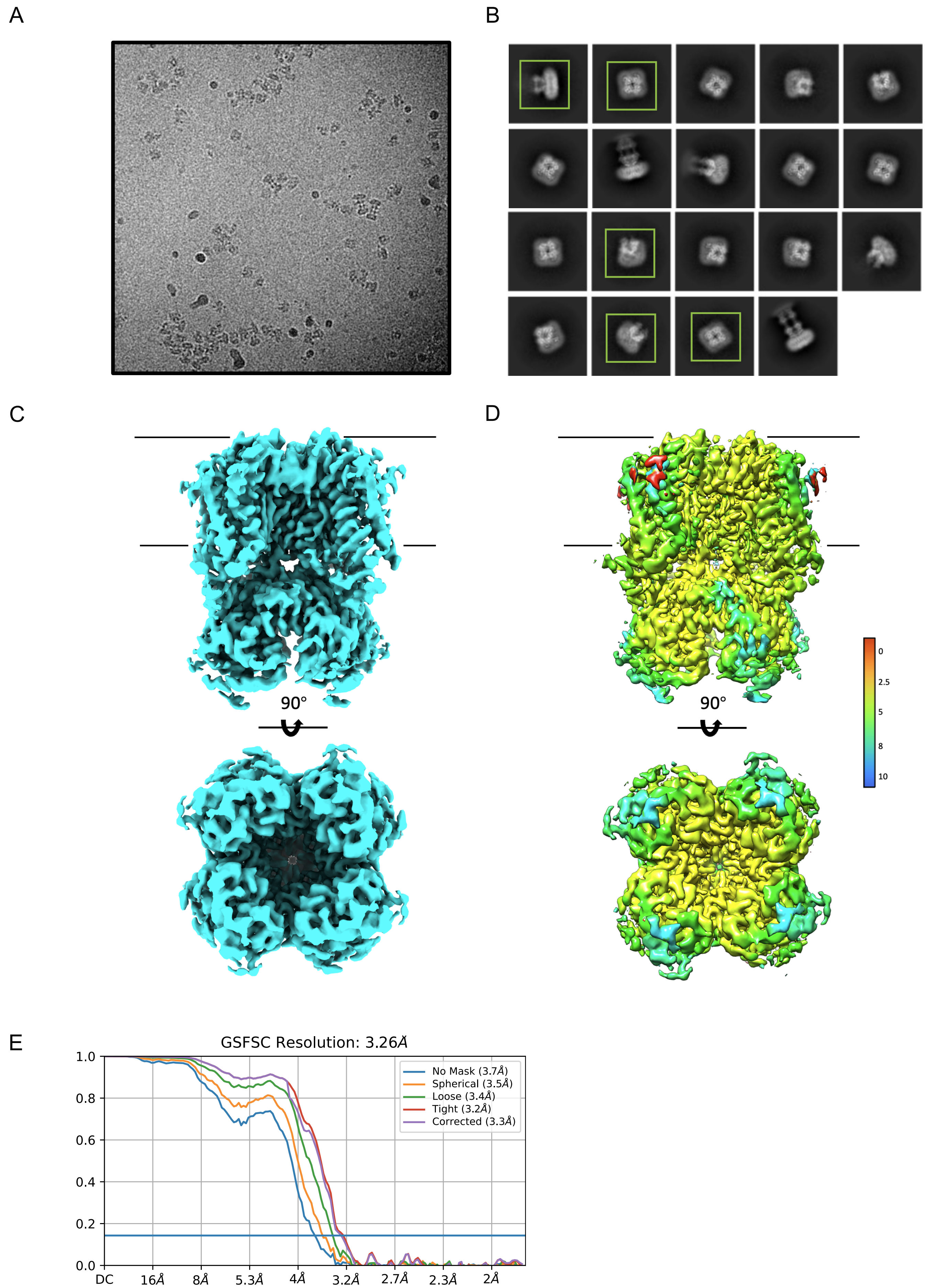

Supplementary Fig. 4 | Single-Particle Cryo-EM of HCN112, cAMP-bound state. A) Representative raw micrograph of the HCN112 in its ligand-bound state with cAMP. B) Selected 2D class averages. Examples of bottom, tilted, and side views are highlighted with green squares. C) HCN112 cAMP-bound density map sharpened with a B-factor of -147.7 Å2. D) Local resolution of density map estimated using Cryosparc_v.0.3 varying between 3.26-2.7 Å and colored with Chimera software. E) Fourier shell correlation (FSC) plots of HCN112 cAMP-bound.

Supplementary Fig. 5

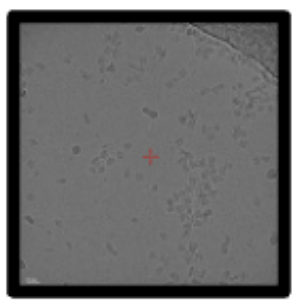

7005 movies

↓

342588
Initial Particles

↓

2D classifications

↓

143502
Particles

↓

Heterogeneous Refinement
v0: 46365 particles
v1: 97137 particles

↓

v1: 97137 particles
NU refinement

↓

Resolution 3.26 Å

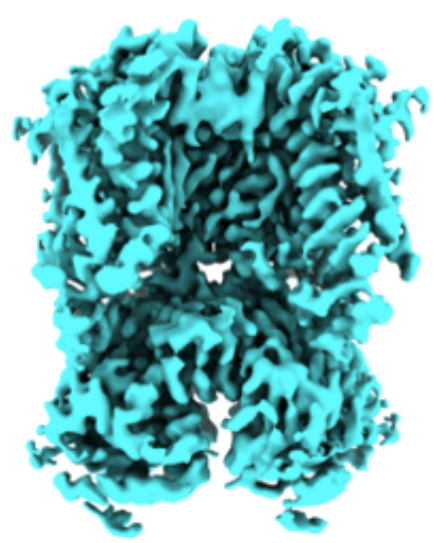

Local resolution 2.7 Å

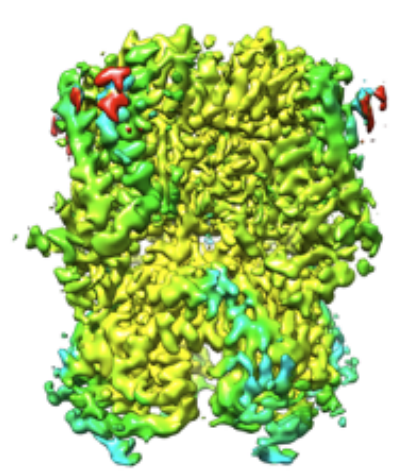

Supplementary Fig. 5 | Single-Particle cryo-EM processing workflow of HCN112, cAMP-bound state. Processing summary for HCN112 cAMP-bound state. After multiple rounds of cleaning by 2D classification the dataset was further processed with 97137 particles as one volume from a heterogeneous refinement step. After NU refinement a resolution of 3.26 Å was achieved, with local resolution of 2.7 Å.

Supplementary Fig. 6

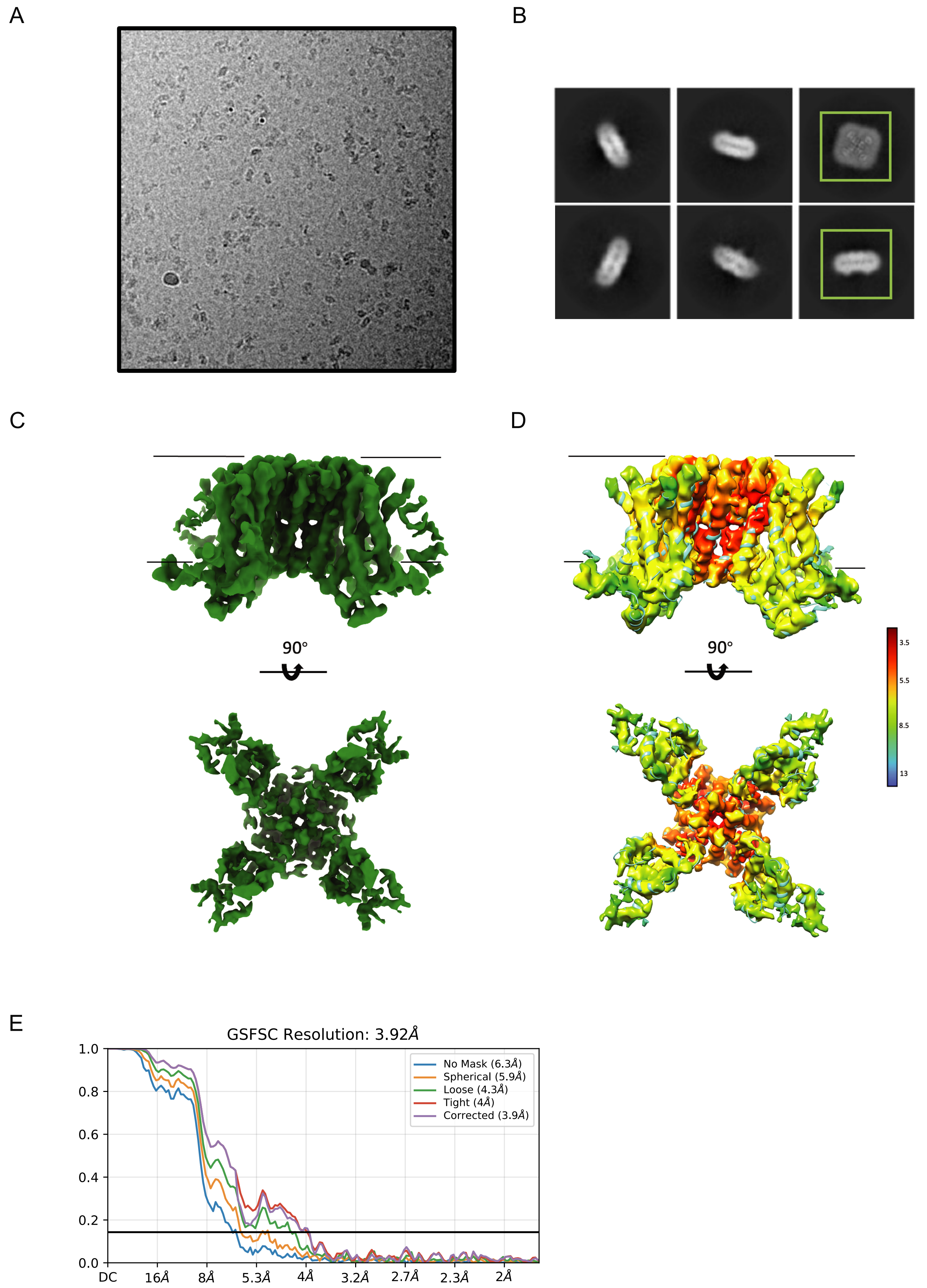

Supplementary Fig. 6 | Single-Particle Cryo-EM of HCN1ΔC. A) Representative raw micrograph of the HCN1ΔC. B) Selected 2D class averages. Examples of bottom and side views are highlighted with green squares.C) HCN1ΔC density map sharpened with a B-factor of -140.8 Å2. D) Local resolution of density map estimated using Cryosparc_v.0.3 varying between 3.92-3.45 Å and colored with Chimera software. E) Fourier shell correlation (FSC) plots of HCN1ΔC.

Supplementary Fig. 7

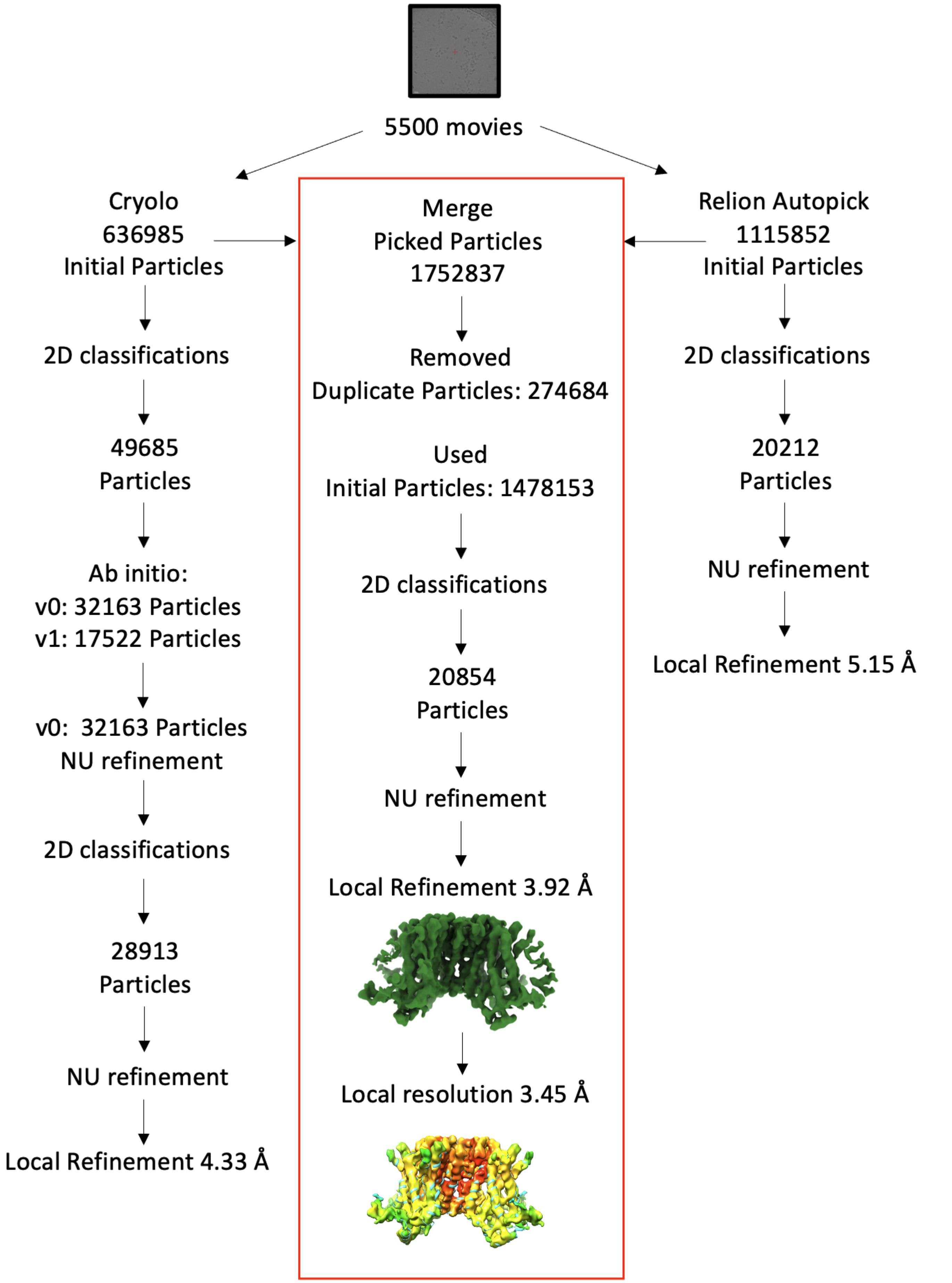

Supplementary Fig. 7 | Single-Particle cryo-EM processing workflow of HCN1ΔC. Processing summary for HCN1 ΔC. Initial processing steps that include picking and multiple rounds of 2D classification were done in parallel, on the unique dataset of 5500 movies, using crYOLO and Relion Autopick. On the two parallel processing, HCN1ΔC could not be resolved to high resolution, therefore picked particles were merged and duplicated removed, leading to 1478153 particles. Subsequent steps of 2D classification and refinement lead to resolution of 3.92 Å with local resolution of density map shown that reached 3.45 Å.

Supplementary Fig. 8

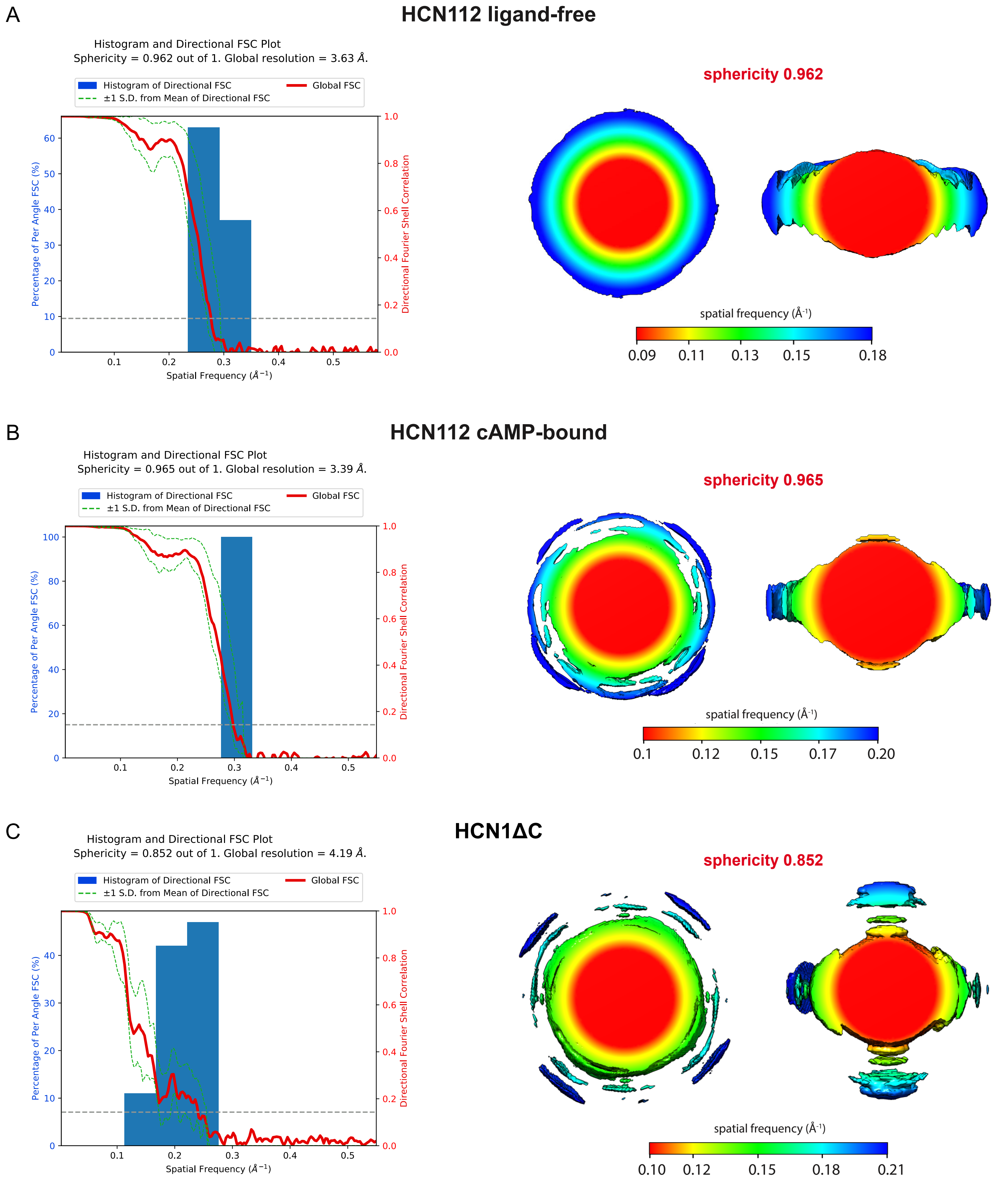

Supplementary Fig. 8 | 3D Fourier shell correlation (3DFSC) analysis of HCN variant cryo-EM maps. Histograms and directional FSC plots illustrating the global half-map resolution and the distribution of directional resolution are shown for each dataset (A–C, left panels). Green dashed lines indicate ±1 standard deviation from the mean directional FSC. Corresponding 3DFSC representations are displayed for top and side views (A–C, right panels). Sphericity values, reported within the histograms and alongside the 3DFSC volumes, are 0.962 for HCN112 ligand-free, 0.965 for HCN112 cAMP-bound, and 0.852 for HCN1ΔC (maximum = 1). The FSC threshold was set to 0.143. (A) HCN112 ligand-free, (B) HCN112 cAMP-bound, and (C) HCN1ΔC. Analyses were performed using the 3DFSC web interface (3dfsc.salk.edu) [ref 1] and visualized in UCSF Chimera.

Supplementary Fig. 9

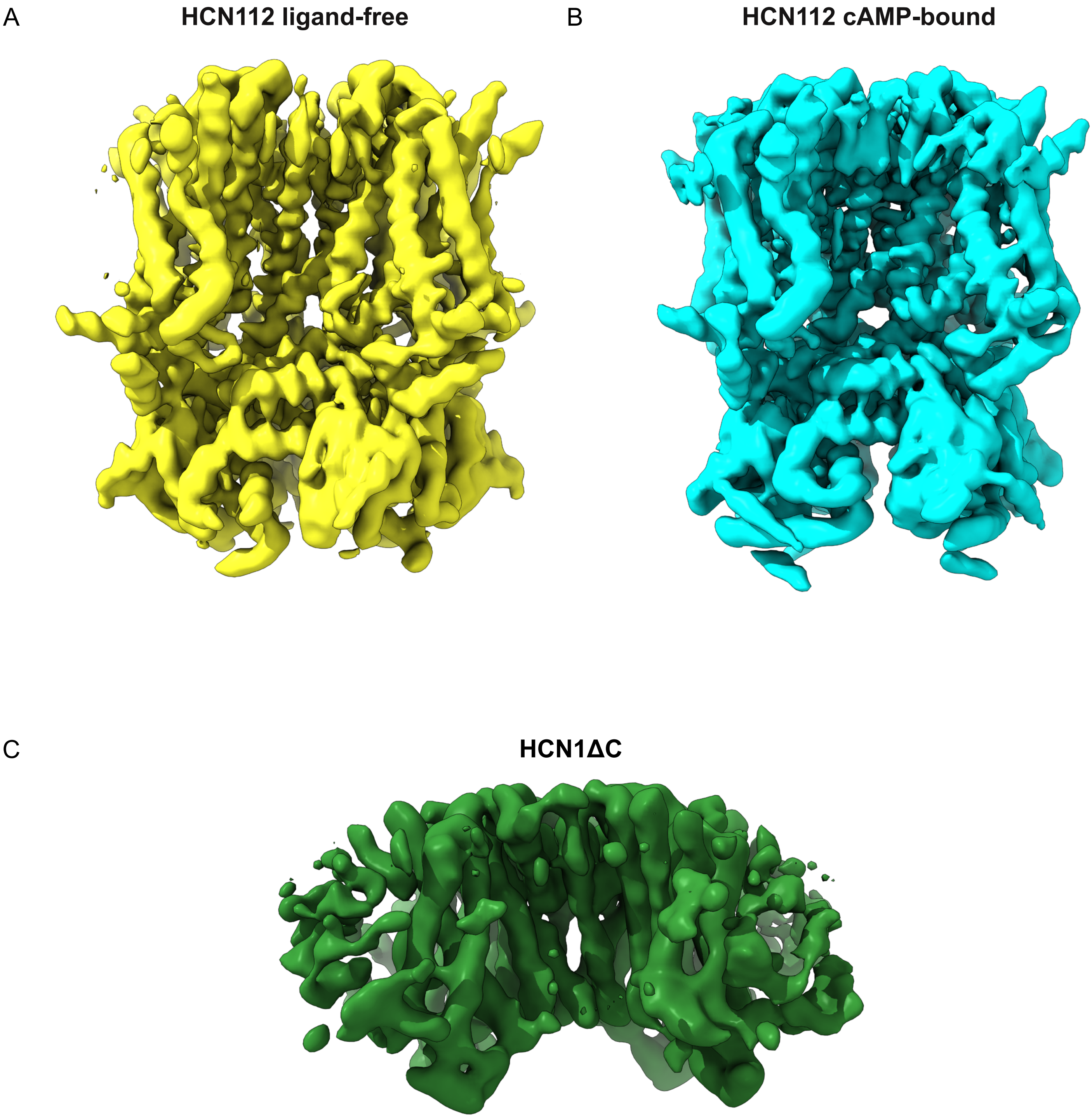

Supplementary Fig. 9 | Cryo-EM raw maps of HCN variants. Unsharpened maps for (A) HCN112 ligand-free, (B) HCN112–cAMP-bound, and (C) HCN1ΔC. Images generated with ChimeraX.

Supplementary Fig. 10

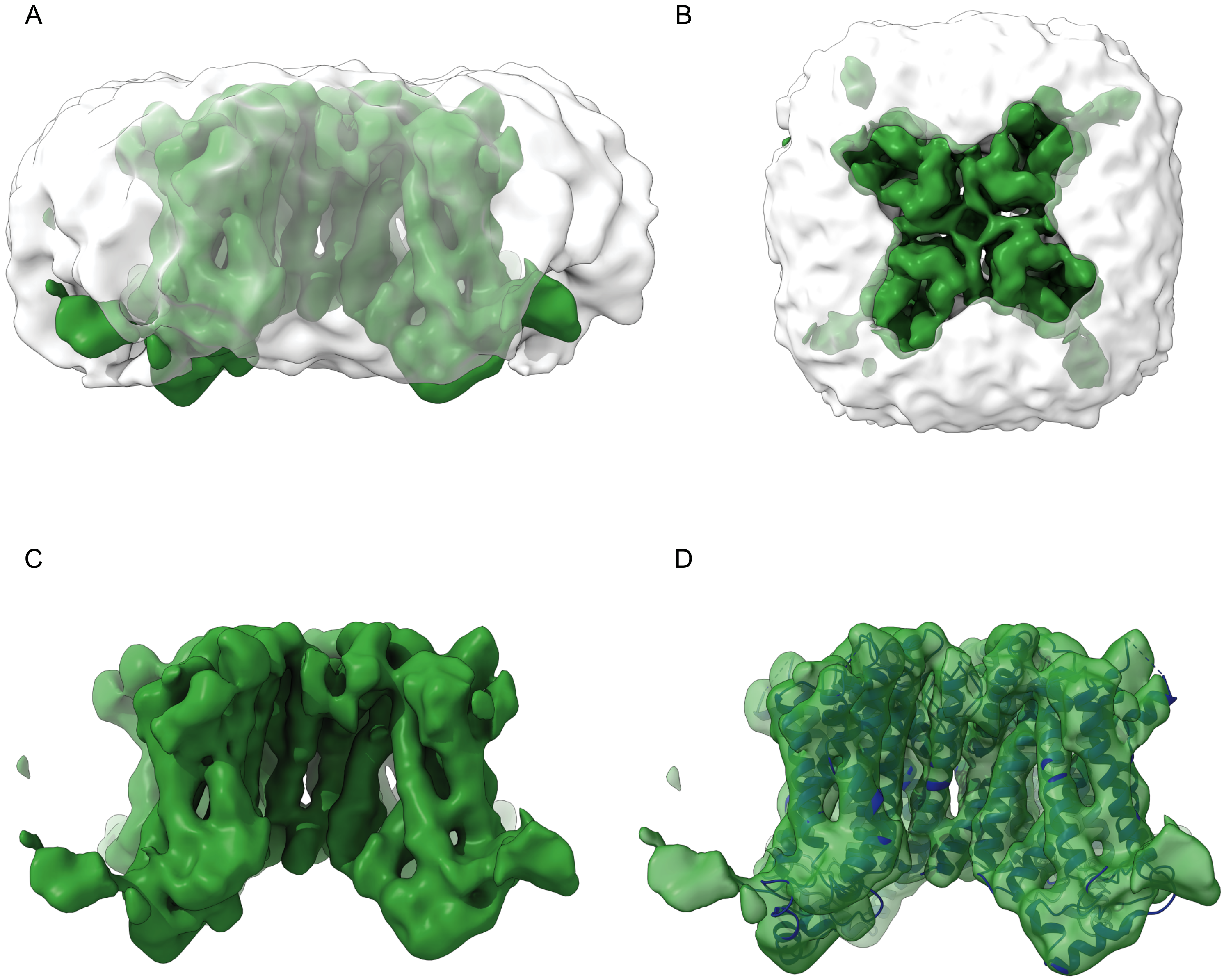

Supplementary Fig. 10 | Cryo-EM map and structure of HCN1Δ. Raw (unsharpened) cryo-EM map of HCN1Δ is shown in green: (A) side view with the detergent micelle (grey, transparent), (B) top view with the detergent micelle, and (C) without the detergent micelle. (D) Overlay of the raw cryo-EM map (green, transparent) and the corresponding HCN1Δ structure (blue). Images generated with ChimeraX.

Supplementary Fig. 11

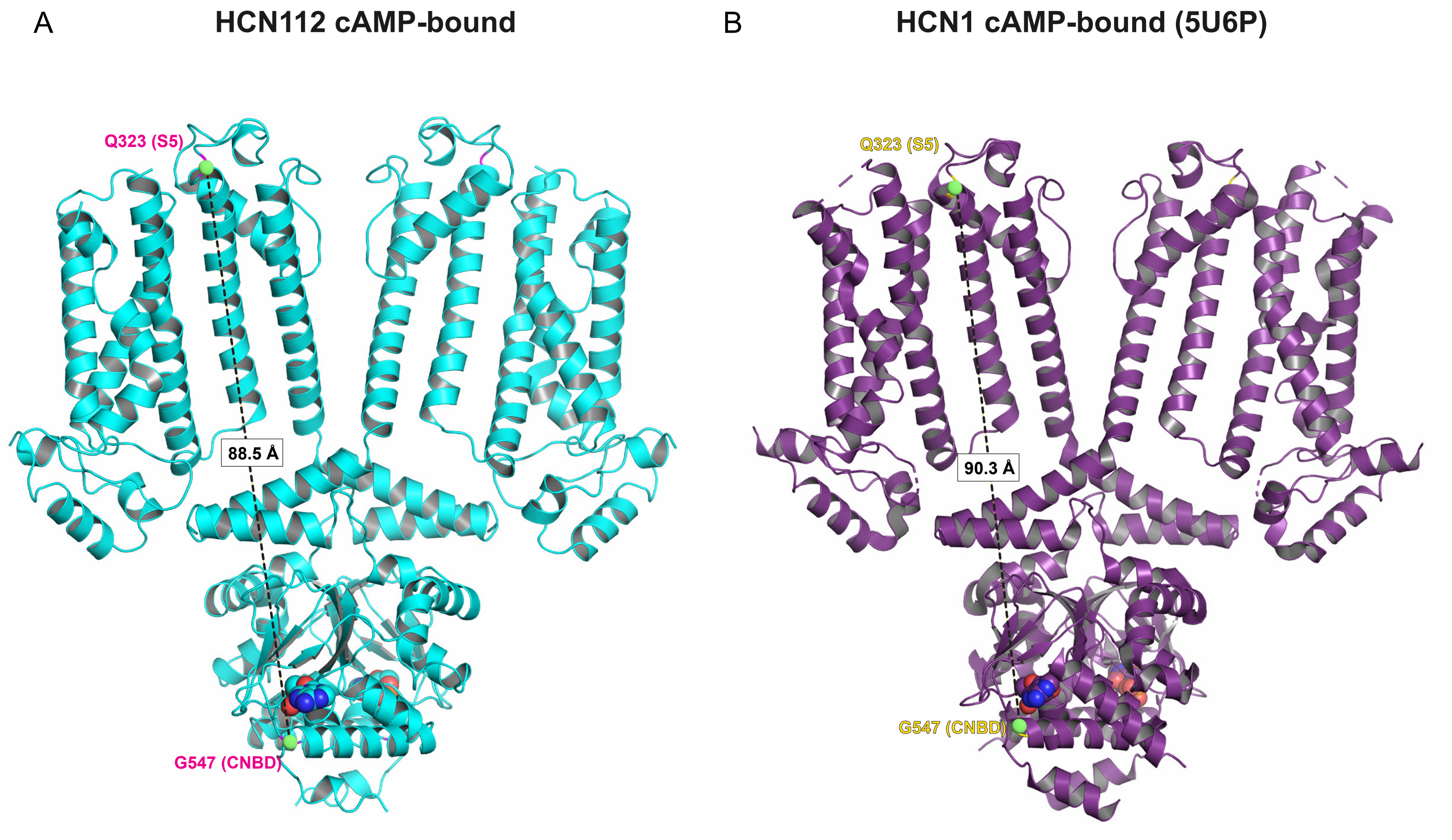

Supplementary Fig. 11 | HCN112 cAMP-bound and HCN1 cAMP-bound. Cartoon representations with only 2 opposing subunits are shown. The green spheres and the dashed line show the distance between the top of S5, residue Q323 and the distal end of the CNBD, residue G547. Images were prepared using PyMOL.

Supplementary Fig. 12

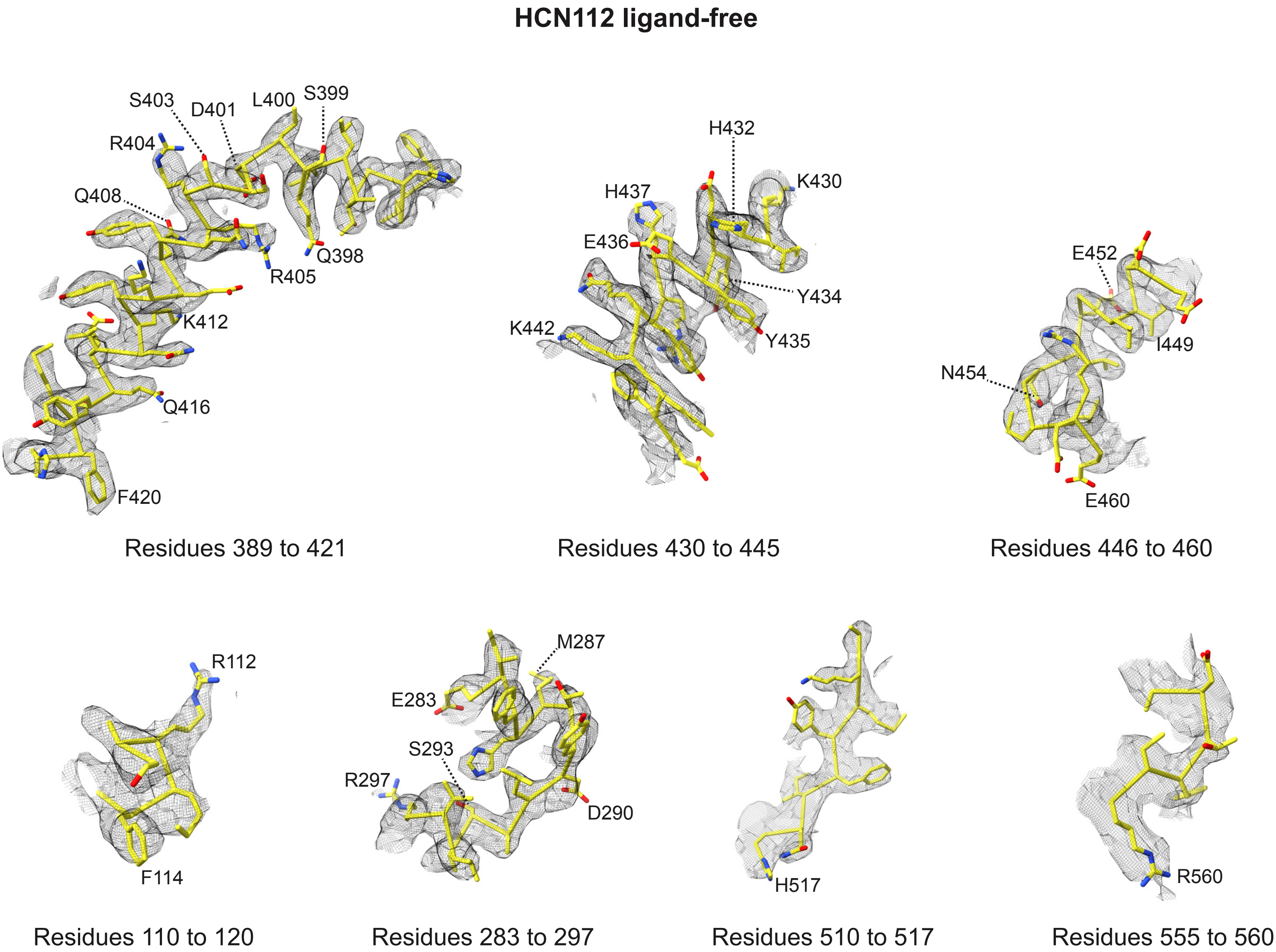

Supplementary Fig. 12 | Cryo-EM density for specific residues of HCN112 ligand-free. Residues from one example subunit (chain A) are shown with their backbones and side chains (in yellow), together with their density (in grey mesh). Important residues discussed on Figure 2 are labelled. Images generated with ChimeraX.

Supplementary Fig. 13

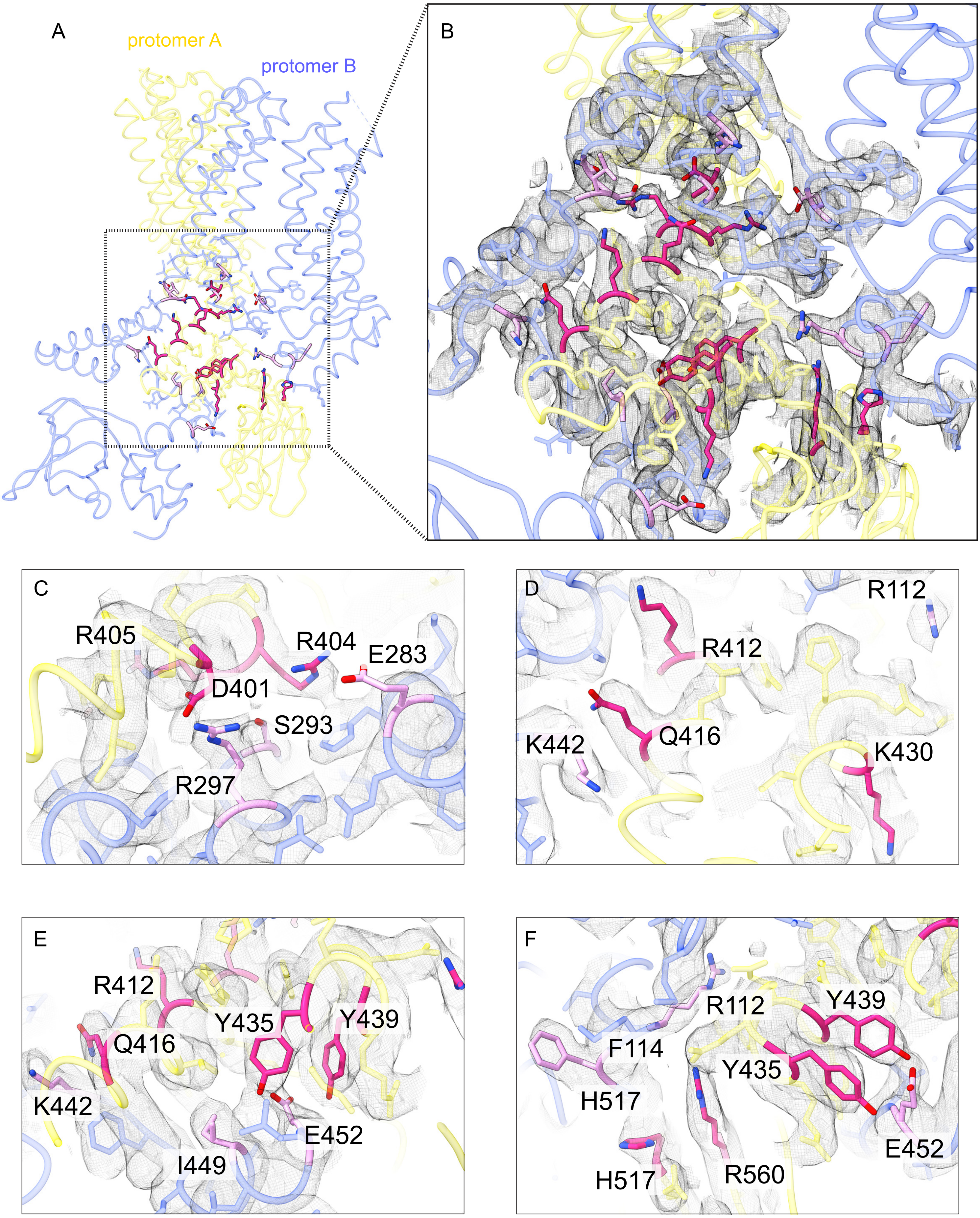

Supplementary Fig. 13 | Cryo-EM density of residues involved in the unique interchain interaction in HCN112 ligand-free. (A) Two HCN112 subunits are shown: protomer A (yellow) and protomer B (blue). Key residues in protomer A are highlighted in pink, and in protomer B in light pink. (B) Zoomed-in view of the region highlighted in panel A, showing the HCN112 structure with the corresponding cryo-EM density for a focused area. (C–F) Close-up views from different orientations highlighting residues involved in the unique interchain interaction, displayed together with their corresponding cryo-EM density. Residues are labeled accordingly. Images generated with ChimeraX.

Supplementary Fig. 14

Cryo-EM density for the C-linker and CNBD

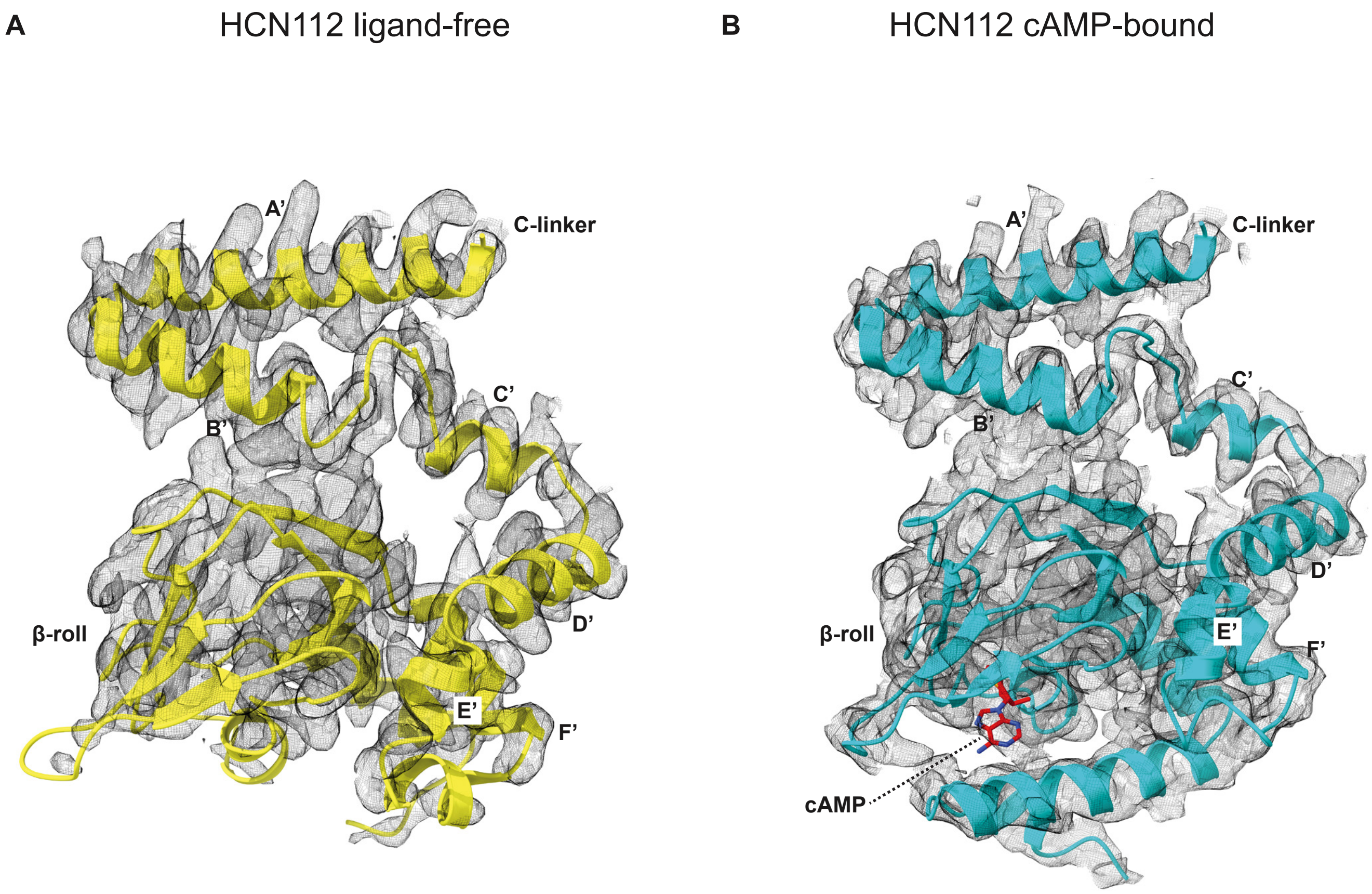

Supplementary Fig. 14 | Cryo-EM density for the C-linker and CNBD domains. A) HCN112 ligand-free (yellow), and B) HCN112 cAMP-bound (cyan). The C-linker and CNBD domains from subunit A are shown as cartoon for both structures, together with the density (in grey mesh). cAMP molecule is shown as atoms and colored red in figure B. The C-linker, helices and β-roll are labelled.

Supplementary Fig. 15

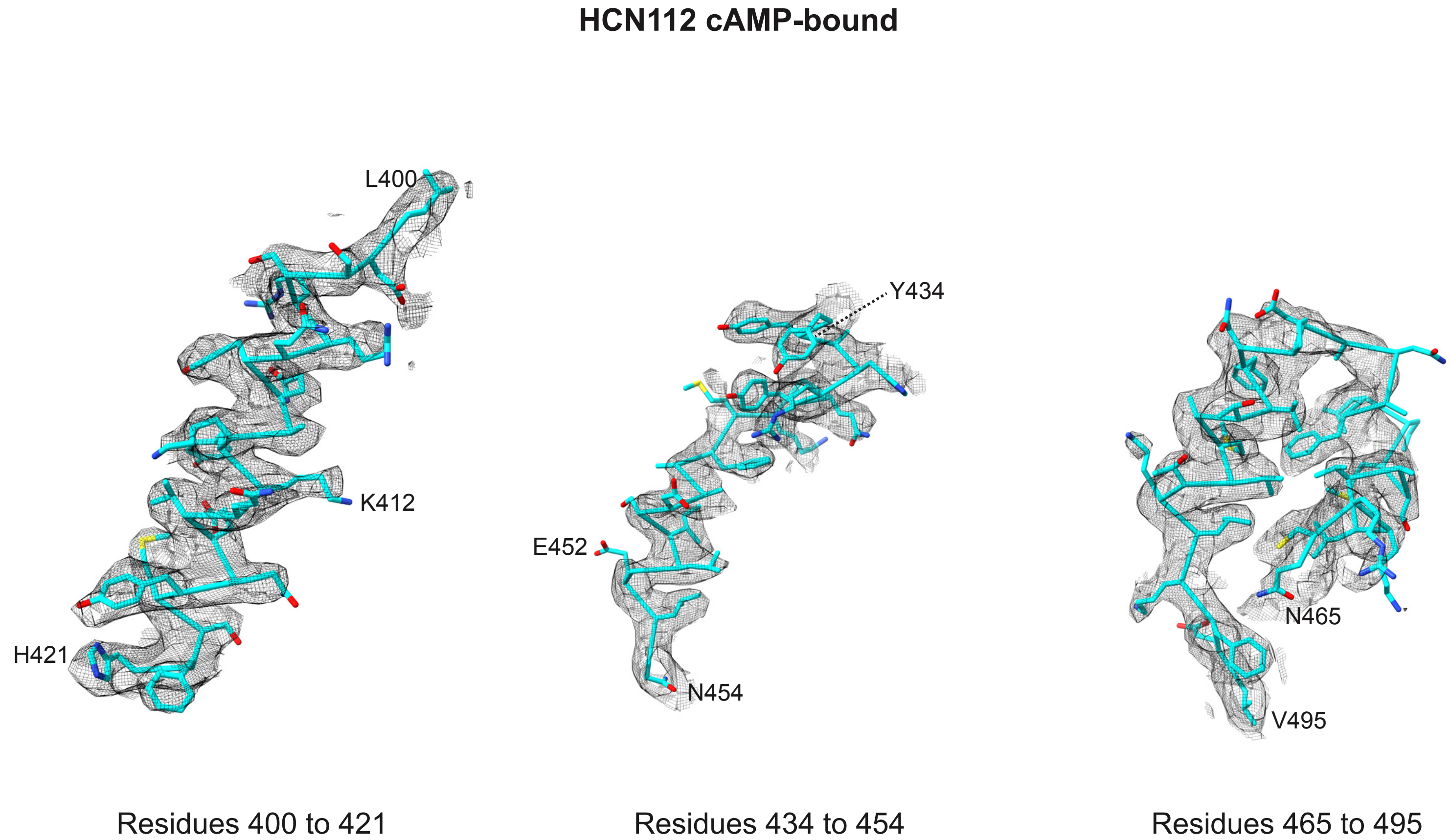

Supplementary Fig. 15 | Cryo-EM density for specific residues of HCN112 cAMP-bound. Residues from one example subunit (chain A) are shown with their backbones and side chains (in cyan), together with their density (in grey mesh). Important residues discussed on Figure 3 are labelled. Images generated with ChimeraX.

Supplementary Fig. 16

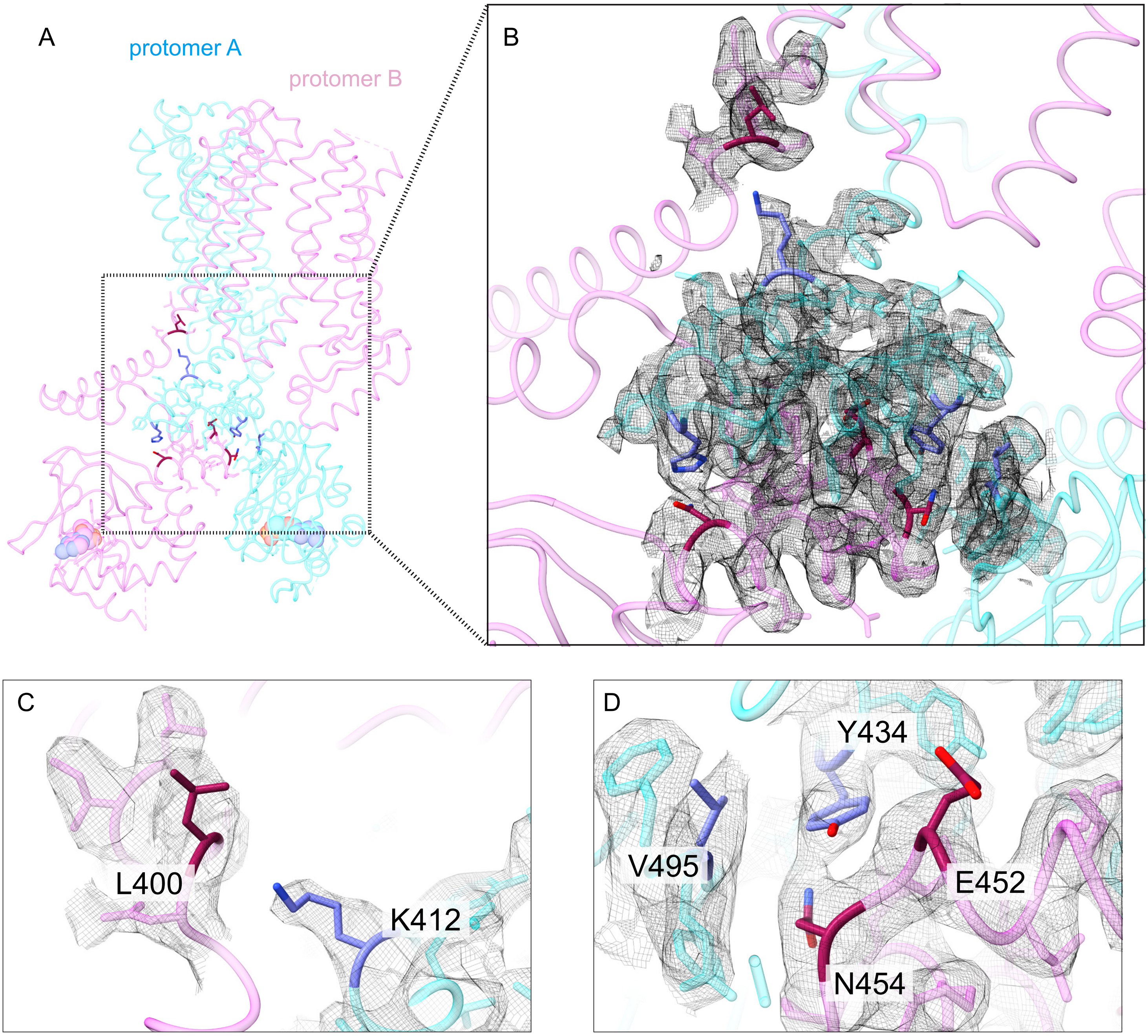

Supplementary Fig. 16 | Cryo-EM density of residues involved in interchain interaction in HCN112 cAMP-bound. (A) Two HCN112 c-AMP-bound subunits are shown: protomer A (cyan) and protomer B (pink). Key residues in protomer A are highlighted in purple, and in protomer B in dark pink. (B) Zoomed-in view of the region highlighted in panel A, showing the HCN112 cAMP-bound structure with the corresponding cryo-EM density for a focused area. (C,D) Close-up views from different orientations highlighting residues involved in the interchain interaction, displayed together with their corresponding cryo-EM density. Residues are labeled accordingly.

Supplementary Fig. 17

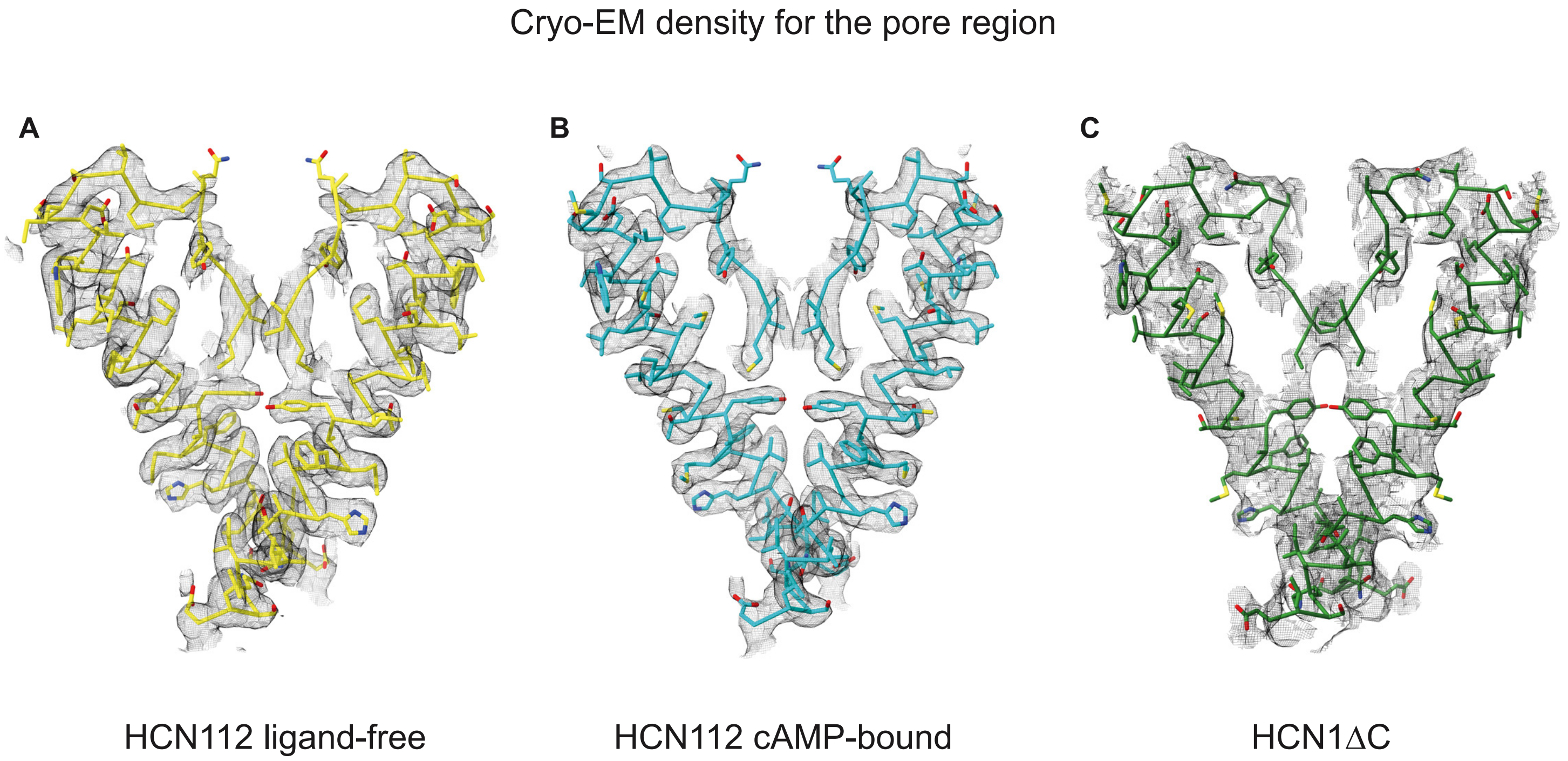

Supplementary Fig. 17 | Cryo-EM density for the pore region of HCN112 ligand-free, HCN112 cAMP-bound, and HCN1ΔC. Pore helices (358 to 401 residues) from subunits A and C are shown with their backbones and side chains (in cyan), together with their density (in grey mesh). Images generated with ChimeraX.

Supplementary Table 1

	#1<br>HCN112 ligand-free<br>(EMDB-53516)<br>(PDB 9R1V)	#2<br>HCN112 cAMP<br>(EMDB-53514)<br>(PDB 9R1T)	#3<br>HCN1ΔC<br>(EMDB-53515)<br>(PDB 9R1U)
**Data collection and processing**			
Magnification	96k	130k	130k
Voltage (kV)	300	300	300
Electron exposure (e–/Å^2)	42.84 (merge 41.42)	32.44	39.81
Defocus range (μm)	-3.2 to -1.6 (0.2 steps)	-3.0 to -1.2 (0.2 steps)	-3.4 to -1.6 (0.2 steps)
Pixel size (Å)	0.81	0.91	0.91
Symmetry imposed	C4	C4	C4
Initial particle images (no.)	370859	342588	1478153
Final particle images (no.)	92412	97137	20854
Map resolution (Å)	3.53	3.26	3.92
FSC threshold	0.143	0.143	0.143
Map resolution range (Å)	3.53-2.9	3.26-2.7	3.92-3.45
**Refinement**			
Initial model used (PDB code)	5U6O	HCN112	HCN112 cAMP
Model resolution (Å)	3.5	3.3	3.1
FSC threshold	0.143	0.143	0.143
Model resolution range (Å)	3.3	3.1	4.0
Map sharpening *B* factor (Å^2)	0.08	0.07	0.12
Model composition	4 chains	4 chains	4 chains
Non-hydrogen atoms	14668	15784	8794
Protein residues	1888	2016	1148
Ligands	-	4CMP	-
B factors (Å^2)			
Protein	61.58	75.72	187.35
Ligand	-	93.96	-
R.m.s. deviations			
Bond lengths (Å)	0.003	0.003	0.003
Bond angles (°)	0.630	0.562	0.787
**Validation**			
MolProbity score	2.06	2.08	2.74
Clash score	16.56	14.89	17.73
Poor rotamers (%)	0.00	1.34	4.73
Ramachandran plot			
Favored (%)	95.14	95.53	92.31
Allowed (%)	4.86	4.47	7.69
Disallowed (%)	0.00	0.00	0.00

Supplementary Table 1 | Cryo-EM data collection and processing, refinement and validation.

Supplementary Movie Legends

Supplementary Movie 1 | Cartoon representation of 2 opposing HCN112 subunits. The morph illustrates the compression movement of the transmembrane domain versus the cytoplasmic domain. The compression is quantified by measuring the distance between the Calpha atom of residue Q323 at the top of the S5 helix and residue G547 at the distal end of the CNBD (indicated as yellow spheres). The compression amounts to ~3.5 Å.

Supplementary Movie 2 | Conformational change of the CNBD upon binding of cAMP in the HCN112 channel. 2 opposing subunits are shown in cartoon representation.

Supplementary Movie 3 | Conformational change of the transmembrane domain (especially S5 and S6) relative to the HCND domain. 2 opposing subunits of HCN1ΔC are shown in cartoon representation.
